# A systematic review and meta-analysis on the epidemiology of work-related musculoskeletal disorders among nurses in Ethiopia

**DOI:** 10.1371/journal.pone.0354484

**Published:** 2026-07-22

**Authors:** Abel Desalegn Demeke, Abraham Dessie Gessesse, Etaferaw Bekele Auddo, Takla Tamir, Alem Bayable, Ababo Demeke

**Affiliations:** 1 Department of Nursing, College of Medicine and Health Sciences, Dilla University, Dilla, Ethiopia; 2 Department of Pediatrics and Child Health Nursing, College of Health Science, Woldia University, Dilla, Ethiopia; Bangladesh University of Engineering and Technology, BANGLADESH

## Abstract

**Background:**

Work-related musculoskeletal disorders (WMSDs) are among the most prevalent work-related disorders affecting individuals across all age groups and professions. Nurses are highly vulnerable due to the nature of their professional duties. However, no comprehensive meta-analysis has been done on the prevalence of WMSDs among nurses. Thus, the purpose of this study was to provide the pooled prevalence and associated factors of WMSDs among nurses in Ethiopia.

**Methods:**

This review was registered with PROSPERO (CRD420251136383). To retrieve relevant studies, various electronic databases such as PubMed, Science Direct, HINARI, and Google Scholar were searched from inception to August 14, 2025. The Joana Briggs Institute critical appraisal tool was used for literature quality assessment. The necessary data were extracted and analyzed with STATA 17. A random-effects model was employed to estimate the pooled prevalence of WMSDs and associated factors while also evaluating heterogeneity and publication bias. Furthermore, a nonparametric trim and fill analysis and leave-one-out sensitivity analysis were conducted.

**Results:**

A total of sixteen studies containing 5982 nurses were included in this study. The pooled prevalence of WMSDs among nurses in Ethiopia was 61.47% (95% CI: 55.08–67.87). Being female (PAOR = 1.37; 95% CI: 1.08–1.75), job stress (PAOR = 1.85; 95% CI: 1.34–2.56), prolonged standing (PAOR = 1.85; 95% CI: 1.29–2.66), and work experience (≥ 5 years) (PAOR = 1.58; 95% CI: 1.08–2.31) were factors significantly associated with WMSDs.

**Conclusion:**

WMSDs among nurses in Ethiopia are considerably high. The finding of our study may warrant attention in workplace risk management such as supportive management and ergonomic training and education, especially for female nurses and those with work experience (≥ 5 years). However, job stress and prolonged standing factors should be interpreted cautiously due to the substantial variability of covariates observed across studies.

## Background

Work-related musculoskeletal disorders (WMSDs) is a term used to express symptoms caused or worsened by work, characterized by damage to muscle, tendon, ligament, nerve tissue, and supporting blood vessels [1]. It is sometimes referred to as cumulative trauma disorder, soft tissue disorders, overuse syndromes, regional musculoskeletal disorders, repetitive motion injuries, and repetitive strain injuries [2]. WMSDs are the main driver of non-communicable disease-related disability burden and one of the most prevalent work-related disorders affecting individuals across all age groups and professions [3,4].

According to the study of Global Burden Disease, the prevalence of musculoskeletal disorders was estimated at 6320 (5510–7220) per 100,000 among people of all age groups in 2020 worldwide [5]. A systematic review and meta-analysis on the global prevalence of WMSDs among nurses found that the overall WMSDs pooled prevalence was 81.1% in the three most affected body areas, such as the lower back (58.5%), neck (45.9%), and shoulders (40.9%) [6]. A meta-analysis conducted in China found that the annual prevalence of WMSDs among nurses was 79% [7]. Another systematic review in Africa estimated the prevalence of WMSDs to range from 15% to 93.5% [8]. However, in low-and middle-income countries (LMICs), particularly in Ethiopia, WMSDs remain less focused due to the emphasis on more pressing and life-threatening health issues like infectious diseases and non-communicable diseases [9].

Among healthcare workers, nurses are the most vulnerable due to the physical demands of their job, which often involve prolonged standing, heavy workloads, frequent patient handling and heavy equipment, and long hours of working and shift rotations [10]. A previously conducted study reported that the prevalence of WMSDs among nurses was found to be higher than in other occupations, such as manufacturing workers and physicians [11,12]. The most commonly reported occupational injuries among nurses include lower back pain [13], neck pain [14], shoulder disorders [15], and upper back pain [2]. Additional conditions such as knee pain, wrist pain, elbow pain, and foot and ankle pain are also prevalent [2,16,17]. These disorders encompass a range of signs and symptoms that can affect health and quality of life, contributing to sick leaves, disability, long-term illness, work limitations, high treatment costs, and absenteeism, thereby imposing a substantial social and economic burden [18,19]. This has serious implications for healthcare delivery, especially in Ethiopia, where the nursing workforce is already strained.

WMSDs among nurses have been considerably associated with various personal, occupational, and environmental factors. These include demographic characteristics such as age, gender, marital status, and body mass index [20–24], as well as professional attributes like years of experience and educational status [20,22,24]. Job-related factors, including long working hours and frequent night shifts, also contribute significantly to the risk [20,23,24]. Working in specific work environments such as operating theaters, medical wards, surgical wards, and intensive care units has been identified as high-risk settings [23,24]. Ergonomic challenges, such as prolonged standing, awkward postures, manual lifting, and repetitive patient-handling tasks, further increase the risk [25,26]. Moreover, lack of assistive devices and insufficient coworker support during procedures are similarly associated with WMSDs [21,22]. Physical fatigue, psychological stress, and underlying health conditions are also responsible [20,24]. According to Sauter and Swanson’s ecological model, office workers’ WMSDs can advance due to psychological variables at work in addition to physical causes. In this approach, psychosocial variables refer to both job and person characteristics that influence job stress, whereas organizational work reflects any work or organizational causes of job stress [27].

Although WMSDs among Ethiopian nurses have been evaluated in a number of primary research studies, the results vary greatly, ranging from 38.1% [26] to 82.9% [28]. The inconsistency across studies, which could hamper the assessment of ongoing intervention efforts and activities. To the best of our knowledge, no compiled meta-analyses have been done on the prevalence of WMSDs among nurses. Therefore, our study aimed to provide the pooled prevalence and associated factors of WMSDs among nurses in Ethiopia. The findings may help inform future research and guide evidence-informed ergonomic and occupational health planning for WMSDs.

## Methods

### Protocol registration and reporting

The International Prospective Register of Systematic Reviews (PROSPERO) has filed the protocol for this systematic review and meta-analysis under registration code CRD420251136383. The protocol was not changed by us.

The updated 2020 Preferred Reporting Items for Systematic Reviews and Meta-Analyses (PRISMA) guideline was used as a template for reporting the present study [29]. All PRISMA 2020 items were addressed; the completed PRISMA 2020 checklist is found in supplemental S1 Table. A flow diagram to demonstrate the process of screening and selecting the included studies was presented, drawing from the PRISMA statement.

### Information sources and search strategy

We systematically searched different electronic databases, such as PubMed, Science Direct, and Hinari, from inception to 14 August 2025 without language restrictions. Moreover, to find additional potentially applicable studies, a Google Scholar search was conducted. Other methods of identifying studies such as reference list screening or manual searching were not performed. The following keywords were used to carry out the search: “musculoskeletal disorder,” “musculoskeletal disease,” “musculoskeletal pain,” “orthopedic disorder,” “musculoskeletal problem,” “musculoskeletal injury,” “musculoskeletal condition,” “nurse,” “health professional,” and “Ethiopia.” Boolean operators such as “OR” or “AND” were used to combine the key words. The details of the search strategy were found in supplemental S2 Table.

### Inclusion criteria

The PECOs (P: Populations; E: Exposures; C: Comparisons; O: Outcomes; S: Study Design) framework was used to settle eligibility criteria. **Populations:** all nurses in any specialty or working unit in Ethiopia; **Exposures:** nurses that experienced WMSDs; **Comparisons:** workers that didn’t encounter WMSDs; **Outcomes:** studies that reported prevalence of WMSDs in at least one part of the body area (neck, shoulder, elbow, wrist/hand, upper back, lower back, hip/thigh, knee, and foot/ankle) in the past one year; **Study design:** a primary research article with an observational study design such as cross-sectional, case-control or cohort; **Publication and Country:** published and unpublished studies conducted in Ethiopia.

### Exclusion criteria

Studies that report disorder of the musculoskeletal system that are not related to the workplace or occupational setting and that do not report the result by differentiating nurses from other health professionals or that are conducted on nursing students were excluded. Additionally, case reports, narrative reviews, editorials, correspondence, abstracts, methodological studies and grey papers, or not completely accessible studies, were deemed ineligible because of issues about inadequate information.

### Study selection process

After all identified articles were imported to EndNote X8, the duplicates were removed. Following this, two authors (ADD & AD) independently read each study’s title and abstract, and then potentially relevant articles were pinpointed. Finally, they separately examined the full text of these articles; as a result, all articles meeting the pre-settled inclusion criteria were chosen. When any disagreement happened between the two authors, the third author reviewed the article, and a decision was made.

### Outcome measurement

The primary outcome of this study was to determine the pooled prevalence of work-related musculoskeletal disorder among Ethiopian nurses. This is calculated by dividing the number of study participants who had experienced WMSDs in at least one part of the body (wrist, elbow, shoulder, neck, low back, upper back, knee, ankle/foot, or thigh) in the past 1 year by the actual sample size, then multiplying by 100. Our secondary outcome focused on identifying factors associated with WMSDs. A pooled adjusted odds ratio (PAOR) was calculated to assess the relationship between WMSDs and their associated factors.

However, included primary studies differed in anatomical scope: some evaluated WMSDs over many body parts, while others reported results limited to a single body location (e.g., low back pain or ankle/foot pain). As a result, the pooled prevalence estimate should be taken as an overall estimate across heterogeneous anatomical definitions because it incorporates studies with varying outcome scopes, which may affect comparability. Where available, we performed subgroup analyses according to the number of body regions assessed to partially account for this heterogeneity in outcome scope.

### Operational definition

Work-related musculoskeletal disorders are defined as the symptoms caused or worsened by work, which are characterized by damage to muscle, tendon, ligament, nerve tissue, or supporting blood vessels and experienced by the participant in at least one part of the body (wrist, elbow, shoulder, neck, low back, upper back, knee, ankle/foot, or thigh) in the past 1 year [30,31].

### Quality assessment and risk of bias

The methodological quality of selected studies was assessed by using the Joana Briggs Institute JBI critical appraisal checklist [32]. To ensure a thorough assessment, two researchers (ADD & ADG) independently evaluated the quality of included studies. In cases where there was disagreement among the researchers, the third authors (EB & TT) resolved the issues. These methodological quality assessment tools have 9 items, each with four possible responses such as yes, no, unclear, and not applicable. The response “yes” indicates the quality is met, and all studies that have 5 and above “Yes” were included in this study [33]. All included studies had met the quality (low risk of bias) (S3 Table) which maintains reliability and increases the validity of our results. However, this designation reflects methodological adequacy relative to the JBI criteria and does not exclude residual bias typical of cross‑sectional designs.

### Data extraction

The following information, such as the author’s name, publication year, sample size, prevalence of WMSDs, measurement tools of each study, and factors associated with WMSDs, was obtained and recorded in a Microsoft Excel spreadsheet independently by two researchers (ADD & AB). Any disagreement that arose between the authors was resolved by the third authors (EB & TT).

### Statistical analysis

A statistical analysis was conducted using STATA software (version 17). Figures and tables were used to show the summarized and descriptive results. We have conducted a meta-analysis to assess the pooled prevalence and determinants of WMSDs. We have estimated the PAOR with their confidence intervals to measure the association. Given the substantial variability of covariates observed across studies, this PAOR estimate should be interpreted as approximate summaries, rather than precise effect estimates. A random-effect model with the methods of DerSimonian and Laird’s was considered to determine the pooled prevalence and determinants of WMSDs among nurses in Ethiopia. The level of heterogeneity across the included studies was depicted by the I-squared (I^2^) index, with the values of <50%, 50–75%, and >75% interpreted as low, moderate, and high heterogeneity, respectively [34]. To address the issue of heterogeneity, we conducted a subgroup analysis by number of body areas assessed, region, sample size, and publication year. A funnel plot and Egger’s test were used to identify the potential presence of publication bias through visual inspection and P-value, respectively. Asymmetrical distribution of the funnel plot and statistical significance (P-value < 0.05) of Egger’s test indicate the presence of publication bias. A nonparametric trim and fill analysis was conducted to address the issue of publication bias. A leave-one-out sensitivity analysis was conducted to identify the effect of a single study on the overall pooled estimate. Finally, the results were reported with confidence intervals of 95% and a significance level of p  <  0.05.

## Results

**Fig 1:**
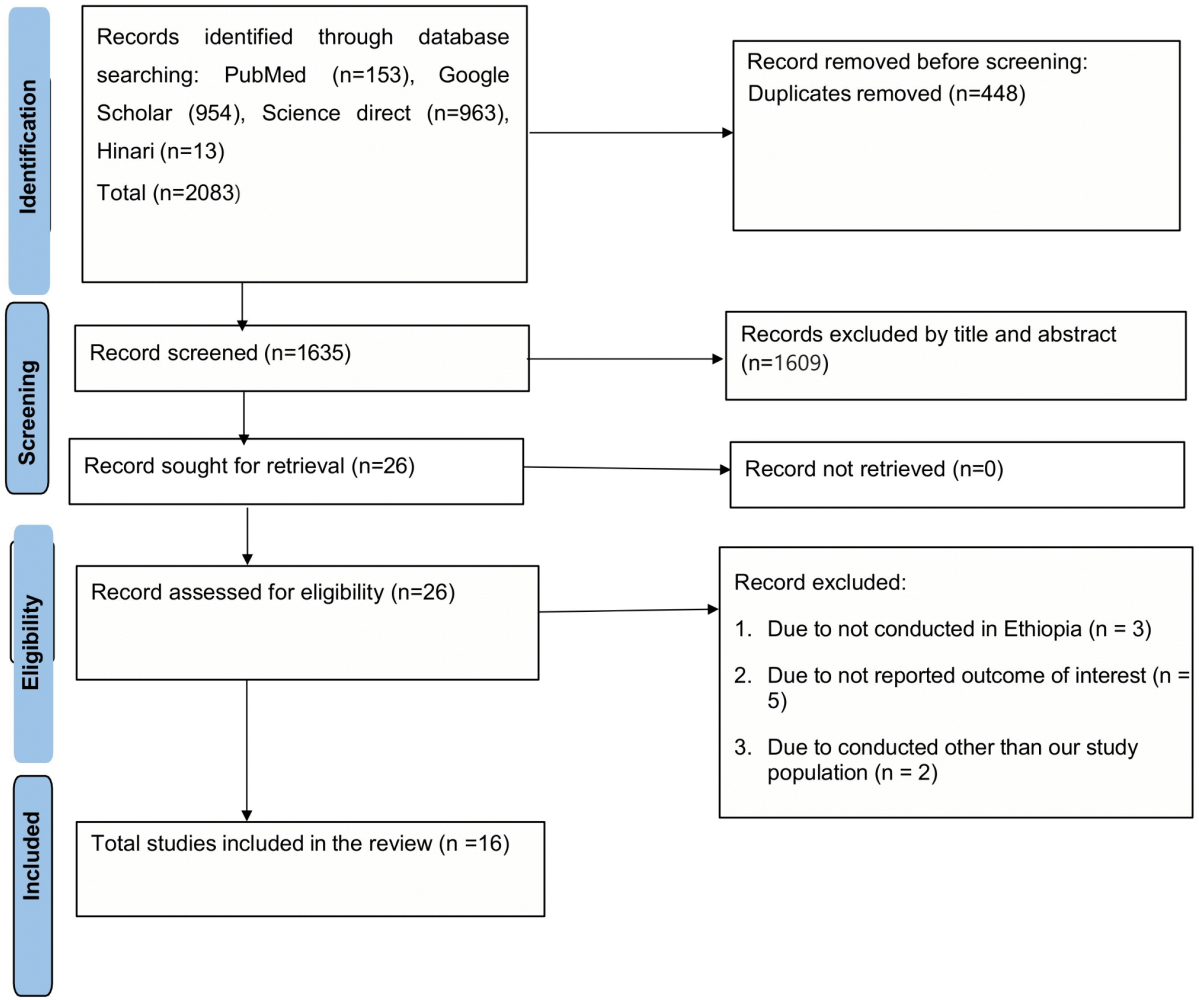
A flowchart of PRISMA showing a selection of study processes for systematic review and meta-analysis on the prevalence of WMSDs and its associated factors among nurses in Ethiopia.

Initially, a total of 2083 studies were obtained from a search of four electronic databases. After eliminating the duplicate, 1635 studies remained. Then, 1609 studies were excluded based on their titles and abstracts because they are out of our scope of interest. Following this, a full text review of 26 studies was assessed for eligibility, and 10 studies were excluded due to different reasons, such as not reporting the outcome of interest, being conducted outside of Ethiopia, and being other than our study population. Finally, a total of 16 studies were found to meet the inclusion criteria and were included in this study by following the guideline of the PRISMA flow diagram (Fig 1).

### Characteristics of the included studies

In this study, a total of 5982 participants from 16 primary studies were included. All of the included studies were conducted by employing a cross-sectional study design and reported the prevalence of WMSDs ranged from 38.1% [26] to 82.9% [28]. Based on the geographical location of the included study, five were conducted in the regional state of Amhara [22,23,35–37], four in Oromia [25,38–40], three in Addis Ababa [28,41,42] and one study from each of the four regions, Sidama [21], SNNPR [43], Harari [26] and Tigray [44]. The sample size among included studies ranged from 120 [40] to 470 [43] (Table 1).

**Table 1:** Characteristics of the included studies for determining prevalence and associated factors of WMSDs among nurses in Ethiopia.

Authors	Publication year	Region	Study design	Sample size	Number of assessed body areas	Measurement tools	Prevalence of WMSDs
Nemera et al. [38]	2024	Oromia	CS	397	Nine	NMQ	73.8
Banga et al. [21]	2024	Sidama	CS	391	One	NMQ	61.9
Regassa et al. [25]	2018	Oromia	CS	301	Nine	DMQ	60.8
Yitayeh et al. [35]	2015	Amhara	CS	389	Nine	NMQ	57.1
Yizengaw et al. [22]	2021	Amhara	CS	394	Nine	NMQ	79
Mijena et al. [26]	2020	Harari and Dire Dewa	CS	404	One	NMQ	38.1
Getie et al. [44]	2021	Tigray	CS	366	One	NMQ	43.7
Tamir Tsehay et al. [23]	2023	Amhara	CS	409	One	NMQ	51.8
Negash et al. [36]	2022	Amhara	CS	423	One	NMQ	52.81
Mekonnen. [39]	2019	Oromia	CS	422	One	NMQ	63.6
Belay et al. [41]	2016	Addis Ababa	CS	430	One	VAS	45.8
Sikiru and Shmaila. [40]	2009	Oromia	CS	120	One	SSQ	60
Yehualaw. [28]	2017	Addis Ababa	CS	448	One	NMQ	82.9
Gashawbeza and Ezo. [43]	2022	SNNPR	CS	470	One	SSQ	54.6
Kore et al. [42]	2021	Addis Ababa	CS	196	One	SSQ	67.2
Tefera et al. [37]	2021	Amhara	CS	422	One	NMQ	76

CS, Cross-Sectional; DMQ, Dutch Musculoskeletal Questionnaire; NMQ, Nordic Musculoskeletal Questionnaire; SSQ, Self-structured Questionnaire; SSPR, South Nation Nationalities and the Peoples Region; VAS, Visual Analog Scale.

### Meta-analysis

#### Overall Prevalence of work-related musculoskeletal disorder among Ethiopian nurses.

**Fig 2:**
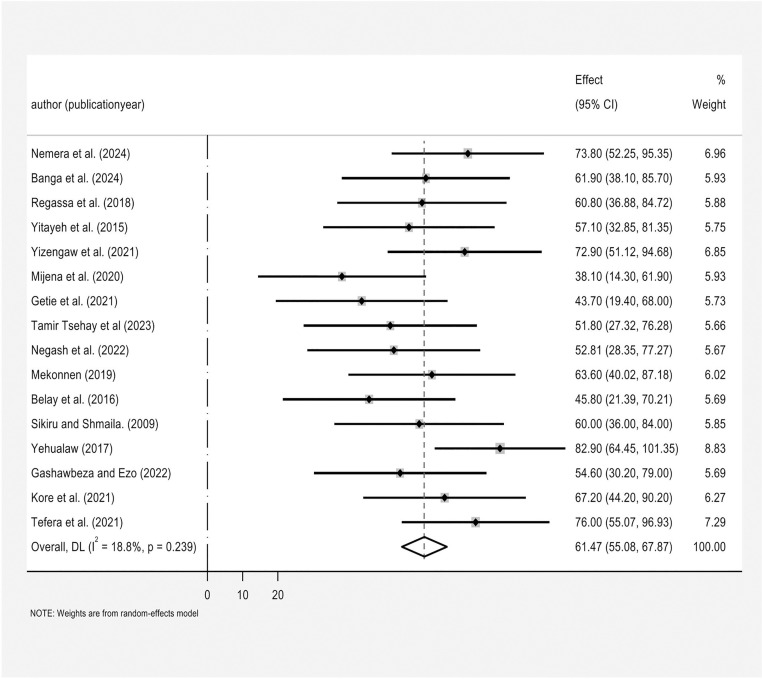
Forest plot showing the pooled prevalence of WMSDs among nurses in Ethiopia.

The estimated pooled prevalence of WMSDs among Ethiopian nurses was found to be 61.47% (95% CI: 55.08–67.87). The level of heterogeneity across the included studies was low, as indicated by the I^2^ index of 18.8% with a P value of 0.239 (Fig 2).

#### Subgroup analysis.

Even though low heterogeneity was depicted between the included studies, we have conducted subgroup analysis by the number of assessed body areas, regions, sample size, publication years, and measurement tool.

**Fig 3:**
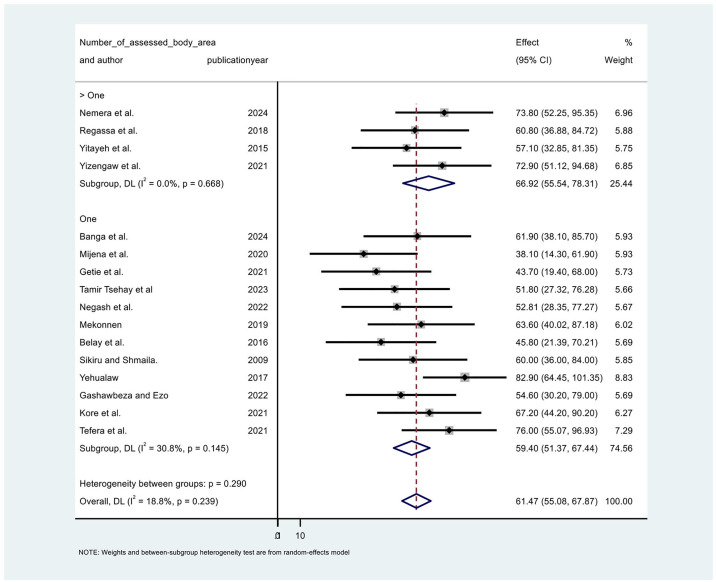
Forest plot showing subgroup analysis by number of body areas assessed for the prevalence of WMSDs among nurses in Ethiopia.

**Fig 4:**
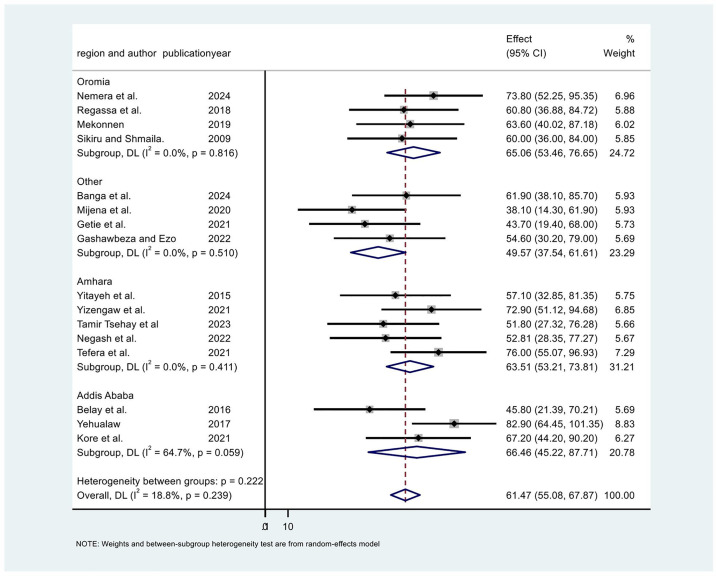
Forest plot showing subgroup analysis by regions for the prevalence of WMSDs among nurses in Ethiopia.

**Fig 5:**
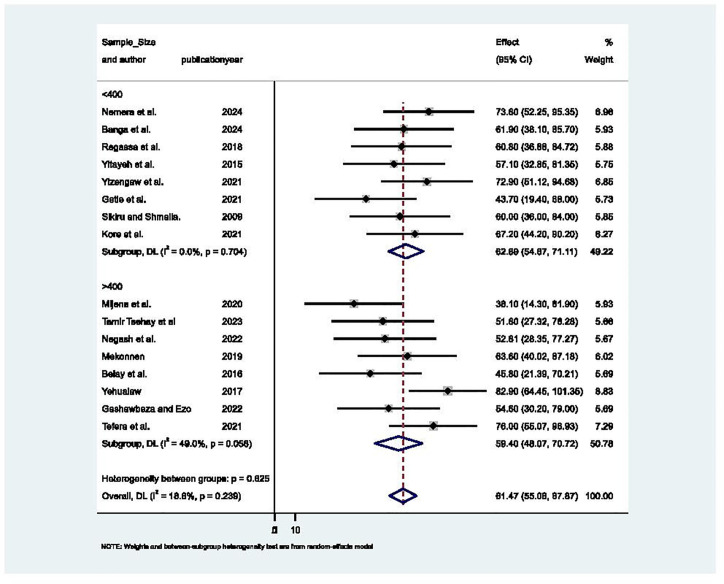
Forest plot showing subgroup analysis by sample size for the prevalence of WMSDs among nurses in Ethiopia.

**Fig 6:**
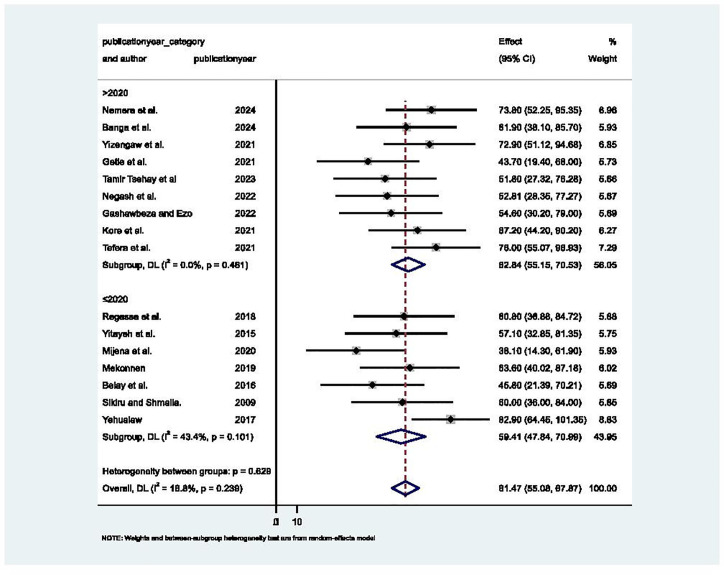
Forest plot showing subgroup analysis by publication year for the prevalence of WMSDs among nurses in Ethiopia.

**Fig 7:**
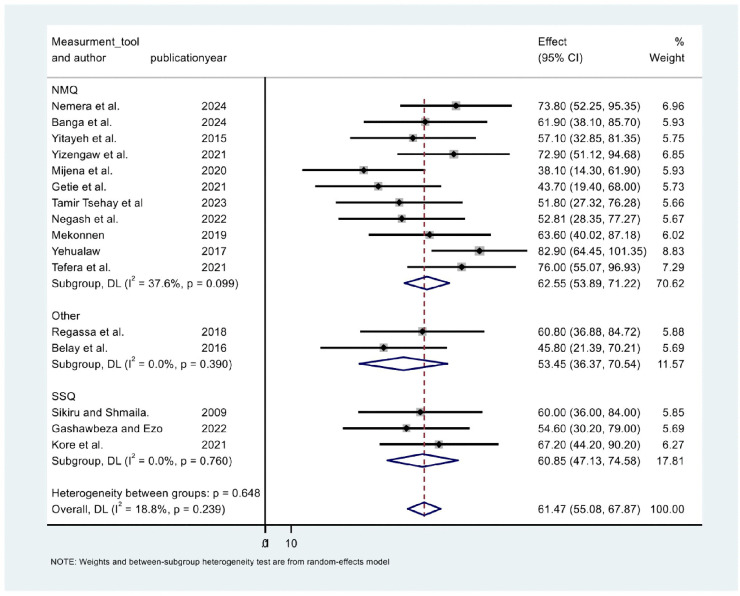
Forest plot showing subgroup analysis by measurement tool for the prevalence of WMSDs among nurses in Ethiopia.

Result of the subgroup analysis showed that the highest prevalence was observed in studies that assessed more than one body area, 66.92% (95% CI: 55.54–78.31, I^2^ = 0.0%) (Fig 3), in the Addis Ababa region, 66.46% (95% CI: 45.22–87.71, I^2^ = 64.7%) (Fig 4), have less than 400 sample size 62.89% (95% CI: 54.67–71.11, I^2^ = 0.0%) (Fig 5), conducted after 2020 year 62.84% (95% CI: 55.15–70.53, I^2^ = 0.0%) (Fig 6), and used Nordic Musculoskeletal Questionnaire (NMQ) measurement tool 62.55% (95% CI 53.89, 71.22, I^2^ = 37.6%) (Fig 7).

#### Publication bias assessment.

**Fig 8:**
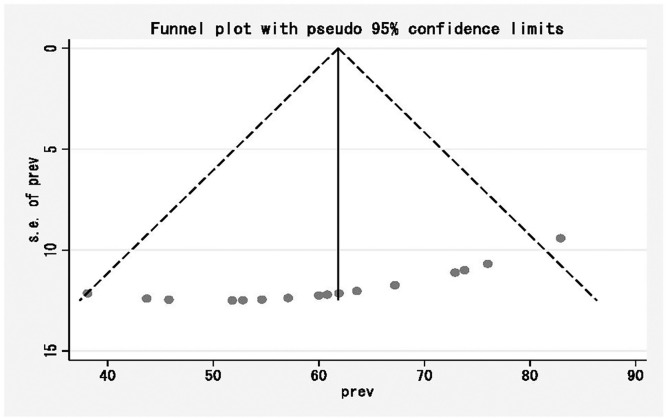
A funnel plot to examine the publication bias of WMSDs among nurses in Ethiopia.

**Fig 9:**
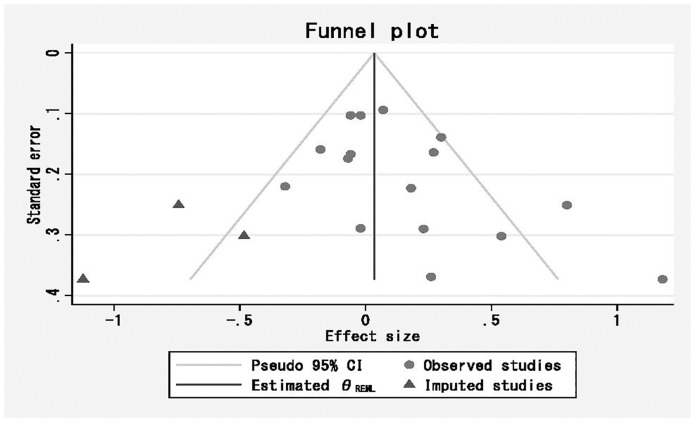
A funnel plot using trim and fill analysis for publication bias of WMSDs among nurses in Ethiopia.

The asymmetrical distribution of the included studies depicted by the funnel plot (Fig 8) and the statistical significance of the p-value (p < 0.001) from Egger’s regression indicate the presence of publication bias. Therefore, to treat this, we have conducted a non-parametric trim and fill analysis, resulting in 19 identified primary studies. After the inclusion of 3 hypothetical missing studies, the funnel plot appeared more symmetrical (Fig 9).

#### Sensitivity analysis.

**Fig 10:**
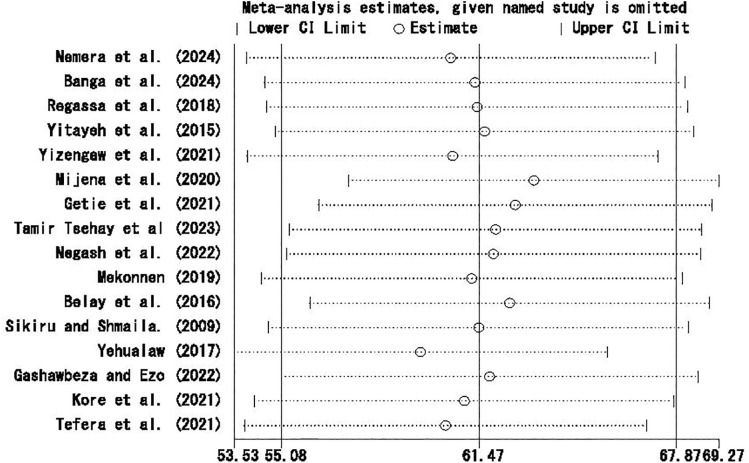
A sensitivity analysis of pooled prevalence for WMSDs among nurses in Ethiopia.

The leave-out-one sensitivity analysis was conducted to assess the effect of a single study on the overall pooled prevalence of WMSDs among Ethiopian nurses. The result showed that the pooled prevalence of WMSDs was not significantly changed by sequential exclusion of any single study, as visually illustrated in (Fig 10), which indicated the robustness of our finding.

### Factors associated with work-related musculoskeletal disorder

A total of 14 variables, such as age, sex, smoking status, working in malposition or awkward posture, bending or twisting during work, assistance from a worker, job stress, job satisfaction, sleep disturbance, working in the same position or static posture, prolonged standing, work experience, working hours per week, and night shift work, were repeatedly presented in at least two included primary studies (Table 2).

**Table 2:** A pooled adjusted odds ratio of factors associated with WMSDs among nurses in Ethiopia.

Variables		Number of study participant	Number of included studies		PAOR (95% CI)	Heterogeneity		
						I^2^	P-value	
Age	20-29	1	1		1	1	1	
	30-39	1678	4		0.81 (0.57-1.16)	0.00%	0.617	
	≥ 40	1678	4		0.73 (0.43-1.25)	7.8%	0.354	
Sex	Male	1	1		1	1	1	
	Female	2463	6		1.37 (1.08-1.75)	60.7%	0.026	
Smoking Status	No	1	1		1	1	1	
	Yes	795	2		1.81 (0.87-3.76)	0.0%	0.849	
Working in a malposition/awkward posture	No	1	1		1	1	1	
	Yes	1480	4		1.29 (0.86-1.95)	75.5%	0.007	
Bending/twisting back during work	No	1	1		1	1	1	
	Yes	1087	3		1 (0.60-1.66)	79%	0.008	
Assistance from a co-worker	Yes	1	1		1	1	1	
	No	839	2		1.86 (0.21-2.84)	0.0%	0.836	
Job stress	Not stressed	1	1		1	1	1	
	Stressed	1235	3		1.85 (1.34-2.56)	70.8%	0.033	
Job satisfaction	Satisfied	1	1		1	1	1	
	Not satisfied	813	2		1.25 (0.82-1.90)	0.0%	0.417	
Sleep disturbance	No	1	1		1	1	1	
	Yes	1225	3		2.75 (0.89-3.99)	89.9%	<0.00	
Working in the same position/static posture	No	1	1		1	1	1	
	Yes	1473	4		1.23 (0.89-1.70)	51.3%	0.104	
Prolonged standing	No	1	1		1	1	1	
	Yes	1205	3		1.85 (1.29-2.66)	91.4%	<0.00	
Work experience	< 5 years	1	1		1	1	1	
	≥ 5 years	1225	3		1.58 (1.08-2.31)	38.6%	0.196	
Working hours per week	< 40	1	1		1	1	1	
	40-47	775	2		1.92 (0.98-3.78)	0.0	0.745	
	≥ 48	775	2		1.59 (0.98-2.59)	65.1%	0.09	
Night shift work	No	1	1		1	1	1	
	Yes	1205	3		1.34 (0.96-1.87)	41.6%	0.18	

1: Reference category; PAOR: Pooled adjusted odds ratio; CI: Confidence interval Note: PAOR should be interpreted as approximate summaries, rather than precise effect estimates, due to substantial variability of covariates observed across studies.

Our study had found sex, job stress, prolonged standing, and work experience were significant factors associated with WMSDs. However, there was no significant association between ages, smoking status, malposition or awkward posture working, worker assistance, job satisfaction, sleep disturbance, static or same posture working, working hours per week, and night shift work with WMSDs.

**Fig 11:**
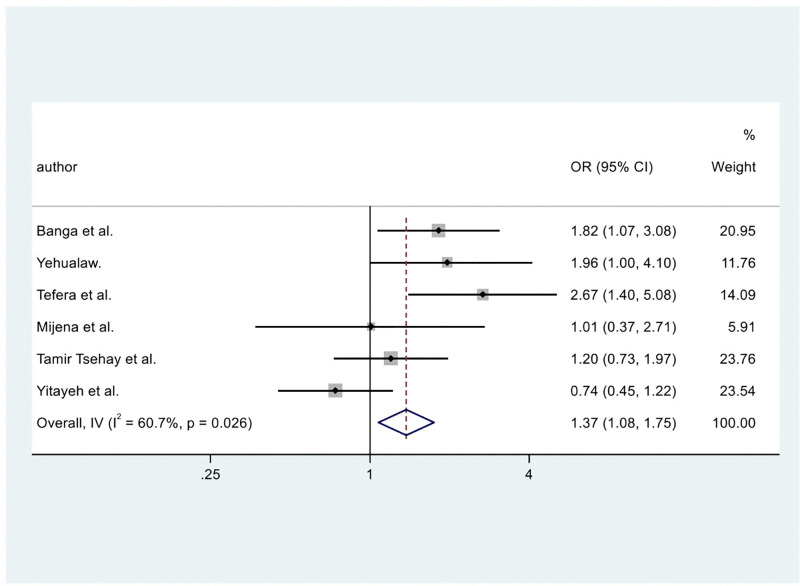
Forest plot showing the association between sex and WMSDs among nurses in Ethiopia.

Six included primary studies [21,23,26,28,35,37] examined the association between sex (female vs male) and WMSDs. Of them, a significant association was found in three included studies. Following the result of this meta-analysis, the PAOR of WMSDs was approximately two times higher among female nurses in comparison to males (PAOR = 1.37; 95% CI: 1.08–1.75; I^2^ = 60.7%) (Fig 11).

**Fig 12:**
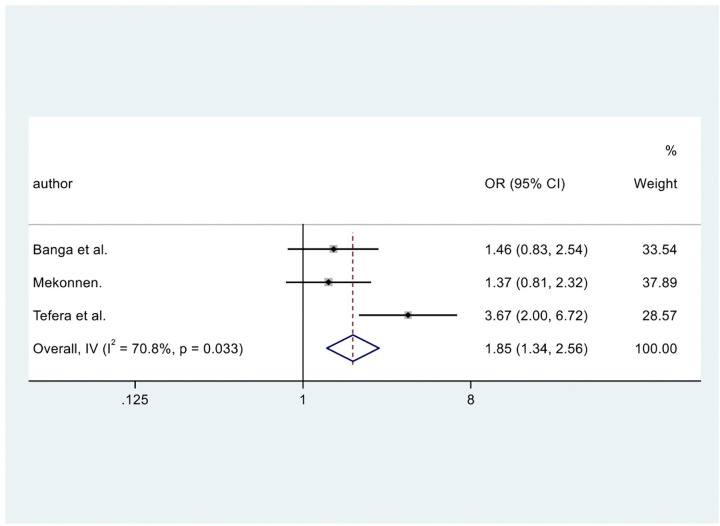
Forest plot showing the association between job stress and WMSDs among nurses in Ethiopia.

Three included studies [21,37,39] determined the association between job stress and WMSDs. Of them, only one study had found a significant positive association. Based on our meta-analysis result, the odds of WMSDs were nearly two times higher among nurses who had job stress as compared to their counterparts (PAOR = 1.85; 95% CI: 1.34–2.56; I^2^ = 70.8%) (Fig 12).

**Fig 13:**
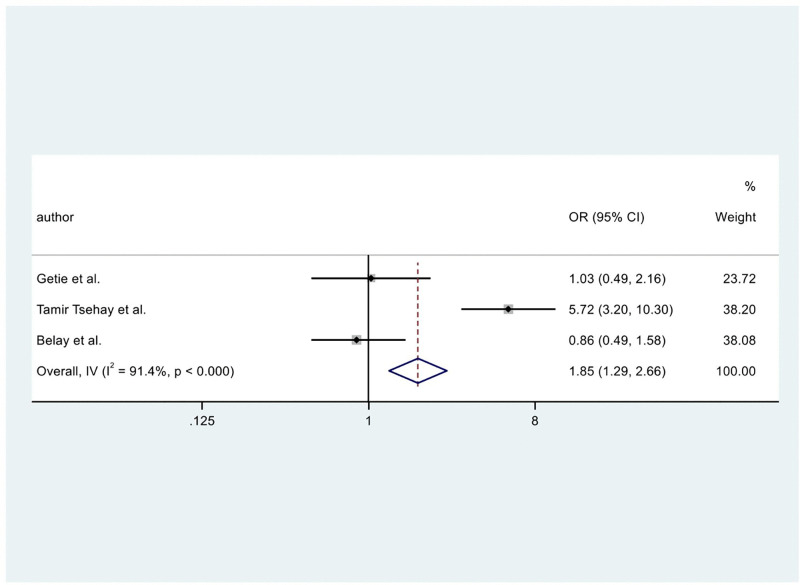
Forest plot showing the association between prolonged standing and WMSDs among nurses in Ethiopia.

Three included studies [23,41,44] examined the association between prolonged standing and WMSDs. Of them, one study found the significant association. This meta-analysis discovered that the odds of WMSDs were approximately two times higher among nurses who had prolonged standing as compared to those who didn’t (PAOR = 1.85; 95% CI: 1.29–2.66; I^2^ = 91.4%) (Fig 13).

**Fig 14:**
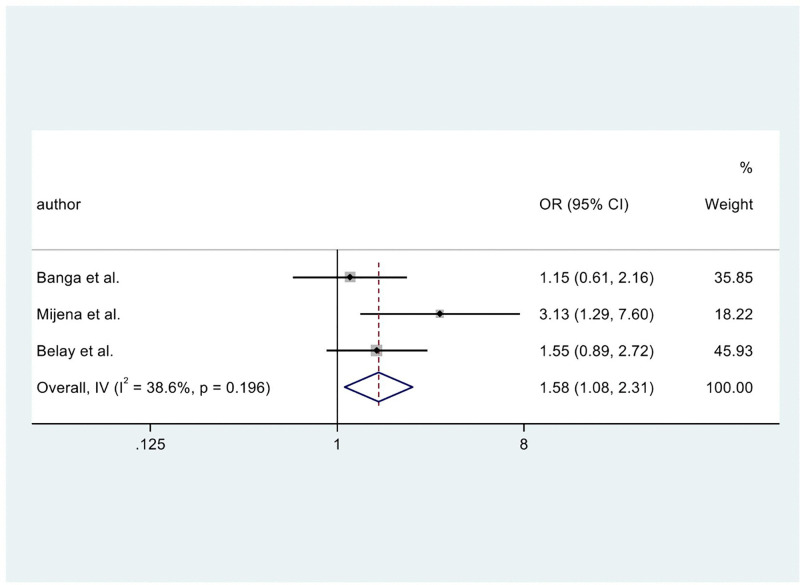
Forest plot showing the association between work experience (≥ 5 years) and WMSDs among nurses in Ethiopia.

Three included studies [21,26,41] examined the association between work experience and WMSDs. Unfortunately, only one of them was found to have a significant association. This meta-analysis discovered that the odds of WMSDs were 1.58 times higher among nurses who had five or more years of experience as compared to those who had less than five years of work experience (PAOR = 1.58; 95% CI: 1.08–2.31; I^2^ = 38.6%) (Fig 14).

## Discussion

To the best of our knowledge, this is the first systematic review and meta-analysis seeking to estimate the overall pooled prevalence of work-related musculoskeletal disorders (WMSDs) and their associated factors among nurses in Ethiopia. A total of 16 articles that fulfilled the inclusion criteria were included and enrolled in the final analysis. Accordingly, the overall pooled prevalence of WMSDs among nurses in Ethiopia was determined to be 61.47% with a substantially low heterogeneity (I^2^ = 18.8%) between included studies.

This finding is comparable with the study that reported the prevalence of low back pain among operating room personnel globally (61.48%) [45], nurses in Italy (57.5%) [46] and Iran (61.48%) [47]. Similarly, our finding is in line with a systematic review study conducted among health care professionals in Africa that reported the prevalence of WMSDs specifically among nurses (65.3%) [48]. Evidence from broader Sub-Saharan Africa also reports generally high prevalence of WMSDs, with prevalence varying across regions [49].

However, notable inconsistencies exist across regions. For instance, prevalence estimates in Pakistan (31.6%) [50], South Africa (38%) [51] and Tunisia (48.1%) [52] are lower. These differences may reflect stronger occupational health systems, improved ergonomic interventions, and greater resource allocation in upper- and lower-middle-income countries compared to Ethiopia. This underscores that WMSDs are not only a biomedical issue but also a reflection of systemic resource constraints, workplace safety policies, and structural inequities in low-income health systems. In the contrary, it is lower as compared to the findings of a systematic review and meta-analysis study conducted among nurses worldwide (77.2%) [53] (81.1%) [6], Europe (87.8%) [54], Asia (84.3%) [13], Vietnam (74.7%) [55], and China (79%) [20]. This inconsistency may be explained by underreporting in Ethiopia, where limited occupational health infrastructure and normalization of pain, may contribute to lower prevalence estimates.

Our study demonstrated that the odds of WMSDs were higher among females as compared to males. This result is supported by studies conducted in Iran [56], India [57], Ethiopia [30]. This might be due to the biological difference between genders after puberty [58]; evidence showed lower or weaker muscle mass [59,60] in females. The reason behind this is that the endogenous testosterone levels is tenfold higher among post pubescent males as compared to females [61], which difference explains the greater muscle mass in males. In addition to biological difference, the observed disparity might be explained by gender-related exposure patterns in the workplace, including domestic workload (e.g., care and housework demands performed mainly by females in addition to employment).

According to this meta-analysis, the odds of WMSDs were two times higher among nurses who had job stress as compared to their counterparts. The evidence generated by this study is in line with the previously conducted studies in China [62], Switzerland [63], Ethiopia [30]. The possible explanation for this is that stress at work leads to fatigability and amplifies the psychological burden; as evidenced, this intensifies the sensation of musculoskeletal symptoms by negatively affecting their pain receptors [64,65]. However, substantial heterogeneity (I² = 70.8%) was observed in this association across studies, which cautions against overgeneralization and suggests that the strength of the relationship between job stress and WMSDs may vary depending on local workplace conditions and psychosocial environments.

The analysis also revealed that a significant association was found between prolonged standing and WMSDs among nurses. Accordingly, the odds of WMSDs were two times higher among nurses who had prolonged standing. This finding is consistent with a systematically reviewed study that examines the association of prolonged standing with musculoskeletal symptoms [66]. In addition to this, the European Agency for Safety and Health at Work also notes that over 60% of workers who stand for long periods report musculoskeletal symptoms as their primary occupational health issue [67]. Prolonged standing leads to reduced blood circulation to the lower limbs, muscle fatigue, and increased joint stress, especially affecting the feet, knees, and lower back [68,69]. It should be noted, however, that our meta-analysis revealed very high heterogeneity (I² = 91.4%) in this association. This variability suggests that the strength of the relationship between prolonged standing and WMSDs may differ across study settings, and findings should be interpreted with caution.

Nurses with five or more years of work experience were found to have 1.58 times the odds of having WMSDs compared to their less experienced counterparts. Different studies have also revealed that having more job experience is associated with WMSDs among nurses [20,24]. This could be explained by the long-term impacts of being exposed to work-related risks, which include repetitive tasks, unfavorable ergonomic circumstances, and insufficient recovery times. In addition, aging-related changes and prolonged exposure to poor ergonomic conditions may also contribute to the increased risk. These mechanisms may interact to amplify the burden of WMSDs over time.

### Implications for practice

The pooled prevalence of WMSDs among nurses in Ethiopia, together with the identified risk factors such as female gender, job stress, prolonged standing, and longer work experience, underscores the need for targeted preventive strategies in nursing practice. These findings suggest that interventions should prioritize ergonomic training, stress management, and workload adjustments. At the same time, the burden of WMSDs must be interpreted within the broader constraints of the healthcare system. Limited resources, high patient-to-nurse ratios, and inadequate ergonomic infrastructure may hinder effective prevention and management, while the absence of strong occupational health policies further exacerbates the challenge. Therefore, addressing WMSDs requires not only clinical and workplace-level interventions but also systemic improvements in staffing, resource allocation, and occupational health programs to ensure sustainable protection for nurses in low-resource settings.

### Strength and limitation of study

The prospective registration of our review protocol in the database of PROSPERO enhances transparency and methodological rigor of the study, as prospective registration of the protocol is crucial for ensuring integrity of research and minimizing reporting bias. Additionally, the PRISMA guidelines were followed in the conduct and reporting of this study. Furthermore, the low level of heterogeneity across the included studies amplifies the analytical result. However, it has its own limitations. First, all of the included primary studies were cross-sectional studies, which may hinder the conclusion of a cause and effect relationship between the variables. Second, it did not consider all regions in Ethiopia due to the limited number of articles, which may limit the generalizability of the findings. Third, trim and fill analysis showed that, after the inclusion of 3 hypothetical missing studies, the funnel plot appeared more symmetrical. As a result, it verified the existence of publication bias; the results should be interpreted carefully by considering this issue. Fourth, studies included in this meta-analysis relied on self-reported WMSDs, which may be prone to recall bias. Fifth, we did not perform reference-list screening or manual searches of identified articles; this may have reduced the completeness of study identification. Six, due to the limited number of included studies per factor, meta-regression was not performed, which would limit statistical power and produce unstable estimates. Lastly, although we acknowledged the high heterogeneity of certain associations in the discussion, we did not perform subgroup or sensitivity analyses to explore potential sources of variability, which may limit the robustness of those pooled estimates.

### Conclusions

This review offers a clear epidemiological understanding of WMSDs among nurses in Ethiopia. The overall pooled prevalence of 61.47% highlights the necessity of formulating precise interventions and health policies for addressing WMSDs in this group of healthcare professionals. In addition, this study found that being female, working on prolonged standing, having job stress, and work experience (≥ 5 years) were significant determinants of WMSDs. The findings may warrant attention in workplace risk management such as supportive management and ergonomic training and education, especially for female nurses and those with work experience (≥ 5 years). However, job stress and prolonged standing should be interpreted cautiously and PAOR as approximate summaries, rather than precise effect estimates, due to substantial variability of covariates observed across studies. Furthermore, this study identifies key areas for future research, which underscore the need for conducting additional primary studies that will address more body areas, as the existence of publication bias was confirmed. Lastly, future researchers should emphasize on qualitative research and longitudinal or context-specific intervention to eliminate WMSDs among nurses.

## Supporting information

S1 Table A checklist of PRISMA 2020. (DOCX)

S2 Table A search strategy. (DOCX)

S3 Table Quality assessment of included studies. (DOCX)

S1 Data Risk of bias assessment of included studies. (XLSX)
