# Supplementary material for: A systematic review and meta-analysis on the epidemiology of work-related musculoskeletal disorders among nurses in Ethiopia
Source: PLoS One. 2026 Jul 22;21(7):e0354484. doi: 10.1371/journal.pone.0354484 (PMC13390853; doi:10.1371/journal.pone.0354484)
Supplement: S2 Table — (DOCX) [file pone.0354484.s002.docx]

| Search terms | database | No of articles | Search date |
| --- | --- | --- | --- |
| ((((((((musculoskeletal disorder[Title/Abstract]) OR (musculoskeletal disease[Title/Abstract])) OR (musculoskeletal pain[Title/Abstract])) OR (orthopedic disorder[Title/Abstract])) OR (musculoskeletal problem[Title/Abstract])) OR (musculoskeletal injury[Title/Abstract])) AND (nurse[Title/Abstract])) OR (health professional[Title/Abstract])) AND (Ethiopia[Title/Abstract]) | Pub med | 153 | 8/5/2025 |
| ("musculoskeletal disorder" OR "musculoskeletal pain") AND ("nurse" OR "health professional") AND ("Ethiopia") | Google scholar | 954 | 8/8/2025 |
| ("musculoskeletal disorder") OR ("musculoskeletal pain") AND ("nurse") OR ("health professional")AND ("Ethiopia") | Science direct | 963 | 8/14/2025 |
| (\("musculoskeletal disorder" OR "musculoskeletal pain"\) AND \("nurse" OR "health professional"\) AND \("Ethiopia"\)) | Hinari | 13 | 8/14/2025 |
