# Supplementary material for: A systematic review and meta-analysis on the epidemiology of work-related musculoskeletal disorders among nurses in Ethiopia
Source: PLoS One. 2026 Jul 22;21(7):e0354484. doi: 10.1371/journal.pone.0354484 (PMC13390853; doi:10.1371/journal.pone.0354484)
Supplement: S3 Table — (DOCX) [file pone.0354484.s003.docx]

| Included studies | Criteria | | | | | | | | | |
| --- | --- | --- | --- | --- | --- | --- | --- | --- | --- | --- |
|  | Q1 | Q2 | Q3 | Q4 | Q5 | Q6 | Q7 | Q8 | Q9 | Total score out of 9 |
| Nemera et al. [[8](#_ENREF_8)] | Y | Y | Y | Y | Y | Y | Y | Y | Y | 9 |
| Banga et al. [[20](#_ENREF_20)] | Y | Y | Y | Y | Y | Y | Y | Y | Y | 9 |
| Regassa et al. [[24](#_ENREF_24)] | Y | Y | Y | Y | Y | Y | Y | Y | Y | 9 |
| Yitayeh et al. [[33](#_ENREF_33)] | N | Y | Y | Y | Y | Y | Y | Y | Y | 8 |
| Yizengaw et al. [[21](#_ENREF_21)] | Y | Y | Y | Y | Y | Y | Y | Y | Y | 9 |
| Mijena et al. [[25](#_ENREF_25)] | Y | Y | Y | Y | Y | Y | Y | Y | Y | 9 |
| Getie et al. [[7](#_ENREF_7)] | Y | Y | Y | Y | Y | Y | Y | Y | Y | 9 |
| Tamir Tsehay et al. [[22](#_ENREF_22)] | Y | Y | Y | Y | Y | Y | Y | Y | Y | 9 |
| Negash et al. [[34](#_ENREF_34)] | Y | Y | Y | Y | Y | Y | Y | Y | Y | 9 |
| Mekonnen. [[36](#_ENREF_36)] | Y | Y | Y | N | Y | N | Y | Y | Y | 7 |
| Belay et al. [[38](#_ENREF_38)] | Y | N | Y | Y | Y | Y | Y | Y | Y | 8 |
| Sikiru and Shmaila. [[37](#_ENREF_37)] | Y | Y | N | N | Y | Y | Y | Y | Y | 7 |
| Yehualaw. [[32](#_ENREF_32)] | Y | Y | Y | Y | Y | Y | Y | Y | Y | 9 |
| Gashawbeza and Ezo. [[40](#_ENREF_40)] | Y | Y | Y | Y | Y | Y | Y | Y | Y | 9 |
| Kore et al. [[39](#_ENREF_39)] | Y | Y | N | Y | Y | Y | Y | Y | Y | 8 |
| Tefera et al. [[35](#_ENREF_35)] | Y | Y | Y | Y | Y | Y | Y | Y | Y | 9 |

Q1; Sample frame appropriate to address the target population?, Q2; Study participants sampled in an appropriate way?, Q3; Sample size adequate, Q4; Study subjects and the setting described in detail? Q5; Data analysis conducted with sufficient coverage of the identified sample?, Q6; Valid methods used for the identification of the condition?, Q7; Condition measured in a standard, reliable way

for all participants? Q8; Appropriate statistical analysis, Q9; Response rate adequate, and if not, was the low response rate managed appropriately? Y; Yes, N; No, Un; Unclear, Na, Not applicable
