# Supplementary material for: A systematic review and meta-analysis on the epidemiology of work-related musculoskeletal disorders among nurses in Ethiopia
Source: PLoS One. 2026 Jul 22;21(7):e0354484. doi: 10.1371/journal.pone.0354484 (PMC13390853; doi:10.1371/journal.pone.0354484)
Supplement: Attachment — Submitted filename: Response to Reviewers.docx [file pone.0354484.s007.docx]

**Editors Comment**

1. **Comment:** Please ensure that your manuscript meets PLOS ONE's style requirements, including those for file naming. The PLOS ONE style templates can be found at https://journals.plos.org/plosone/s/file?id=wjVg/PLOSOne_formatting_sample_main_body.pdf and <https://journals.plos.org/plosone/s/file?id=ba62/PLOSOne_formatting_sample_title_authors_affiliations.pdf>

**Response:** Thank you for your feedback. We have thoroughly reviewed our manuscript and ensured that it meets all of PLOS ONE's style requirements, including those related to file naming conventions. We appreciate your guidance and have utilized the provided templates to align our submission accordingly.

2. **Comment:** Please update your submission to use the PLOS LaTeX template. The template and more information on our requirements for LaTeX submissions can be found at http://journals.plos.org/plosone/s/latex.

**Response:** Thank you for your suggestion. We have updated our submission to utilize the PLOS LaTeX template as per your requirements. We appreciate your guidance in ensuring our manuscript meets the necessary standards.

3. **Comment:** Please upload a new copy of Figures 10 - 13 as the detail is not clear. Please follow the link for more information:  <https://journals.plos.org/plosone/s/figures>

**Response:** Thank you for your valuable feedback regarding Figures 10-13. We appreciate your input, as it greatly enhances the quality of our manuscript. We have uploaded a new copy of Figures 11-14, as the previous details were not clear. Please note that due to the addition of a new figure (Fig 7, subgroup analysis by measurement tool), the order of the figures has changed. Consequently, what were originally Figures 10-13 are now Figures 11-14.

4. **Comment:** Please include a new copy of Table S3 in your manuscript; the current table is difficult to read. Please follow the link for more information: https://journals.plos.org/plosone/s/tables

**Response:** Thank you for your feedback. We have revised Table S3 to enhance its readability by using shorter abbreviations for the questions and providing a clear description below the table. We appreciate your guidance in improving our manuscript.

5. **Comment:** If the reviewer comments include a recommendation to cite specific previously published works, please review and evaluate these publications to determine whether they are relevant and should be cited. There is no requirement to cite these works unless the editor has indicated otherwise.

**Response:** Thank you for your guidance. We appreciate your emphasis on the importance of relevant citations. However, we would like to clarify that the reviewer did not recommend any specific previously published works for us to cite.

**Reviewers comment**

**Reviewer #1**

1. **Comment:** Title and Abstract
   Strengths
   • The objective is clear: estimating pooled prevalence and associated factors of WMSDs among nurses in Ethiopia.
   • Key results (prevalence, associated factors) are concisely summarized.
   Limitations
   • The abstract may be overly long; it reads more like a truncated full manuscript than a standard abstract.
   • Key elements like registration of the review (e.g., PROSPERO number) are missing.

**Response:** Thank you for your positive feedback on the strengths of our manuscript. We have revised our abstract to better align with standard formatting. While the PROSPERO registration number was previously included at the end of the abstract, we have now moved it to the methods section for improved clarity and visibility. Additionally, we have included a statement regarding the assessment of the quality of the literature.

2. **Comment:** Introduction
   Strengths
   • Identifies a clear knowledge gap: lack of compiled meta-analytic evidence on WMSDs in Ethiopian nurses.
   • Provides justification due to high vulnerability of nurses.
   Limitations
   • Insufficient context: Does not explain global WMSD prevalence or compare Ethiopia to other regions.
   • Does not explicitly state a theoretical framework guiding which factors were expected to be associated.

**Response:** Thank you for your constructive feedback on the strengths of our manuscript. We have now included an explanation of the global prevalence of WMSDs and described the constraints faced in low- and middle-income countries, specifically in Ethiopia. Additionally, we have incorporated a theoretical framework (model) that explores the factors alongside those previously listed.

3. **Comment:** Methods

Strengths
• Multiple reputable databases searched (PubMed, ScienceDirect, HINARI, Google Scholar).
• Inclusion of published and unpublished studies reduces publication bias.
• Use of standard analytical elements: random-effects model, heterogeneity assessment (I²), funnel plot, Egger’s test, trim-and-fill, sensitivity analysis.

**Response:** Thank you for your thoughtful comments regarding the strengths of our manuscript! I truly appreciate your acknowledgment of the comprehensive methodology we've employed in our analysis.
Limitations and Concerns
a. No PRISMA Compliance Mentioned
The description should explicitly state adherence to PRISMA 2020 guidelines, including a flow diagram.

**Response:** Thank you for your valuable feedback regarding PRISMA compliance. While we previously mentioned it in the methods section, we appreciate your suggestion and have now explicitly stated our adherence to the PRISMA 2020 guidelines, including the flow diagram.
b. Search Strategy Not Fully Described
Missing:
o Search string(s)
o Language restrictions
o Search date ranges (only end date given)
o Screening process (number of reviewers, resolution of disagreements)
Without these details, replication is difficult.

**Response:** Thank you for your constructive feedback regarding the search strategy. We previously noted that no language restrictions or time bounds were applied in the inclusion criteria. For greater clarity, we have now relocated this information to the "Information Sources and Search Strategy" section. Additionally, we clarified that the studies included were from inception to August 14, 2025, with a specific search conducted from August 5 to August 14. Furthermore, the details of the search strategy can be found in Supplementary Table S2.

Regarding the screening process, we have outlined the number of reviewers and the resolution of disagreements in the "Study Selection Process" subsection. We appreciate your input, which has significantly improved the clarity of our manuscript.
c. Study Quality Assessment Not Reported
• No mention of quality or bias assessment tools (e.g., Newcastle–Ottawa Scale).
• The robustness of included studies cannot be evaluated.

**Response:** Thank you for your reminder regarding the quality assessment of the studies. However, we have clearly described this in the "Data Extraction, Study Quality, and Risk of Bias Assessment" subsection. Specifically, we assessed the methodological quality of the selected studies using the Joanna Briggs Institute (JBI) critical appraisal checklist. We appreciate your attention to this important aspect, as it ensures the robustness of our findings.
d. Lack of Data Extracted Variables
The description does not specify:
o How WMSD was defined across studies
o Whether data extraction was done independently by two reviewers
o Adjusted vs. crude effect sizes

**Response:** Thank you for your valuable concerns regarding the data extraction variables. However, we have previously specified in the inclusion criteria that WMSD is defined as studies reporting the prevalence of WMSD in at least one body area (neck, shoulder, elbow, wrist/hand, upper back, lower back, hip/thigh, knee, and foot/ankle) within the past year. Additionally, we have provided an operational definition. In the "Outcome Measurement" subsection, we clarified that a pooled adjusted odds ratio (AOR) was calculated to assess the relationship between WMSDs and their associated factors. Furthermore, we indicated that data extraction was conducted independently by two researchers (ADD & AB), and any disagreements were resolved by a third author (EB & TT), as detailed in the "Data Extraction, Study Quality, and Risk of Bias Assessment" subsection.
e. No Handling of Variation Between Studies
• WMSD definitions, measurement tools, and anatomical regions may differ widely.
• No subgroup analyses (e.g., by region, measurement tool, year of study), which could help explain heterogeneity.

**Response:** Thank you for highlighting the handling of variation between studies. We addressed the variation in WMSD definitions, and anatomical regions in our predefined inclusion criteria, which specify studies reporting the prevalence of WMSD in at least one body area (neck, shoulder, elbow, wrist/hand, upper back, lower back, hip/thigh, knee, and foot/ankle) within the past year, as well as in the operational definition. We conducted subgroup analyses based on the number of body areas affected by WMSDs. Additionally, we previously performed subgroup analyses by region and publication year. In response to your constructive recommendation, we have now included subgroup analyses by measurement tool as well. We appreciate your input, which has contributed to the robustness of our analysis.

4. **Comment:** Results
   Strengths
   • Provides pooled sample size and number of included studies.
   • Reports pooled prevalence with confidence intervals.
   • Identifies significant associated factors with pooled odds ratios.
   Limitations
   a. High Heterogeneity Likely
   • Although I² is mentioned, its value is not reported.
   • Prevalence studies typically show high heterogeneity; without subgroup/exploratory analyses, results may be unreliable.

**Response:** Thank you for your concern regarding heterogeneity. The I² value is indeed mentioned in the previously submitted manuscript under the "Meta-Analysis" subsection, where it is reported as 18.8%. We also conducted subgroup analyses based on region, sample size, publication year, and the number of anatomical areas. In light of your insightful feedback, we have additionally performed subgroup analyses by measurement tool. We appreciate your input, which has enhanced the rigor of our analysis.
b. No Visuals Reported
• Forest plot, funnel plot, trim-and-fill output, and PRISMA flow diagram are essential but not mentioned or described.
c. Limited Examination of Associated Factors
• Only four factors identified; unclear if others were tested (e.g., BMI, working hours, shift work, patient-handling practices).
d. No Sensitivity Analysis Results Reported
• Stated as conducted but results not summarized.

**Response:** Thank you for your feedback. The forest plot, funnel plot, trim-and-fill analysis, and PRISMA flow diagram were indeed described in the first section of the results and the publication bias assessment. Additionally, sensitivity analysis result was summarized or reported clearly under sensitivity analysis sub section. Moreover, A total of 14 variables were examined in this study, including age, sex, smoking status, working in awkward positions, bending or twisting during work, assistance from a colleague, job stress, job satisfaction, sleep disturbance, prolonged static postures, work experience, weekly working hours, and night shifts. These variables are detailed in the accompanying **Table 2**. Out of these, only four—sex, job stress, prolonged standing, and work experience—were found to have a significant association with work-related musculoskeletal disorders (WMSDs). Thank you again for bringing these points to our attention. Your insights are invaluable for improving the clarity and comprehensiveness of the study.

5. **Comment:** Discussion
   Strengths
   • Conclusion aligns with findings.
   • Suggests practical implications (e.g., focus on reducing stress and standing time).
   Limitations
   • Does not compare pooled estimates with global or African data.
   • Does not address:
   o Potential reverse causation (cross-sectional designs dominate this field).
   o Measurement bias (self-reported outcomes).
   o Differences in workplace ergonomics across studies.

**Response:** Thank you for your valuable feedback on the discussion section. We appreciate your insights regarding its strengths and limitations. We acknowledge the points raised about the comparison with global and African data, potential reverse causation, measurement bias related to self-reported outcomes, and the variations in workplace ergonomics across studies. These aspects have been addressed in the revised manuscript

6. **Comment:** Conclusion
   Strengths
   • Acknowledges high prevalence and identifies actionable factors.
   Limitations
   • Recommendations are generic and lack specificity (e.g., ergonomic programs, workload redesign, staffing ratio improvement).
   • Does not address research implications, such as the need for longitudinal or intervention studies.

**Response:** Thank you for your thoughtful comments regarding the conclusion of the study. We appreciate your recognition of the strengths. In response to your feedback on the limitations, we have revised the recommendations to include more specific strategies, such as the implementation of ergonomic training, improvements in staffing ratios, proper body mechanics etc. Additionally, we have incorporated a discussion on the research implications, emphasizing the need for longitudinal or intervention studies to further explore these issues. Your insights have greatly contributed to enhancing the clarity and depth of our conclusions. Thank you once again for your valuable feedback.

7. **Comment:** Overall Strengths of the Study
   • Addresses an important occupational health issue.
   • Uses meta-analysis to synthesize evidence valuable for policymaking.
   • Includes multiple databases and unpublished studies.
   • Demonstrates methodological awareness (heterogeneity, publication bias).
   Overall Limitations and Areas for Improvement
   Methodological Limitations
   • Insufficient reporting on search strategy and risk-of-bias assessment.
   • Unclear adherence to PRISMA guidelines.
   • No subgroup or meta-regression analyses.
   • Unclear handling of inconsistent definitions of WMSD.
   • High heterogeneity likely undermines pooled prevalence reliability.
   Analytical Limitations
   • Effect estimates may be confounded due to lack of adjusted ORs.
   • No weighting or discussion of study quality.
   Interpretation Limitations
   • Overgeneralizes findings without accounting for workplace variability across Ethiopia.

**Response:** Thank you for your thorough evaluation of the study's strengths and limitations. We truly appreciate your insights on the methodological and analytical aspects, as well as the interpretation limitations. We have taken your feedback to heart and have addressed the issues raised, including enhancing the reporting on the search strategy and risk-of-bias assessment, clarifying adherence to PRISMA guidelines, and discussing the handling of inconsistent definitions of WMSD in the revised manuscript. Thank you again for your constructive comments

8. **Comment:** Summary
   The study provides useful pooled estimates of WMSDs among nurses in Ethiopia, showing a high prevalence (61.47%) and identifying significant associated factors. However, methodological weaknesses especially incomplete reporting of search strategy, lack of study quality assessment, unclear heterogeneity handling, and limited discussion of contextual factors reduce confidence in the pooled estimates. Strengthening transparency, performing subgroup analyses, and adhering strictly to PRISMA standards would significantly improve the study’s rigor and credibility.

**Response:** Thank you for your thoughtful summary and feedback on the study. We appreciate your recognition of the useful pooled estimates and associated factors identified. We acknowledge the methodological weaknesses mentioned, specifically regarding the reporting of the search strategy, study quality assessment, handling of heterogeneity, and discussion of contextual factors. We have made revisions to address these issues, aiming to strengthen transparency, perform subgroup analyses, and adhere more closely to PRISMA standards in the revised manuscript. Thank you once again for your valuable input

**Reviewer #2**

1. **Comment:** Although a PROSPERO registration number is provided, the manuscript does not clearly describe whether the review was conducted strictly according to the registered protocol. Pls. clarify deviations from the registered protocol and explicitly state adherence to the PROSPERO plan.

**Response:** Thank you for your valuable feedback. We appreciate your attention to detail. We have clarified in the manuscript that the study was conducted in strict accordance with the registered protocol.

2. **Comment:** The authors report searching multiple databases; however, the manuscript lacks: Full electronic search strategies, Search strings for each database, Justification for the use of Google Scholar

pls. provide complete search strategies (preferably as supplementary material) to ensure reproducibility, as required by PRISMA 2020.

**Response:** Thank you for your valuable feedback. We have already included the complete search strategies in the supplementary material of the previously submitted manuscript, and we have now added a statement indicating which supplemental file contains the details of the search strategies. In response to your insights, we have also added a justification for the use of Google Scholar.

3. **Comment:** While observational studies were included, the methodology does not sufficiently describe: the specific quality appraisal tool used and How risk of bias influenced interpretation of pooled results. Pls. clarify the tool used and discuss how study quality may have affected the meta-analysis findings.

**Response:** Thank you for your valuable feedback regarding the methodology section. We appreciate your attention to detail. In response to your comments, we have clarified the specific quality appraisal tool used and discussed how the risk of bias may have influenced the interpretation of the pooled results. This addition aims to enhance the transparency and rigor of our study.

4. **Comment:** Substantial heterogeneity is expected in prevalence meta-analyses; however, the manuscript does not adequately explore potential sources of heterogeneity beyond reporting I². pls. consider additional subgroup or meta-regression analyses (e.g., by region, study setting, or assessment tool) or explicitly justify their absence.

**Response:** Thank you for highlighting this important consideration. We appreciate your reminder regarding heterogeneity in prevalence meta-analyses. In the previously submitted manuscript, we have already conducted subgroup analyses by region, number of anatomical areas, sample size, and publication year. Currently, we have now included subgroup analyses by measurement (assessment tool) to address potential sources of heterogeneity more comprehensively.

5. **Comment:** The manuscript reports pooled odds ratios for associated factors; however, the language occasionally implies causality. Pls. revise interpretations to clearly reflect associations, acknowledging the limitations of cross-sectional observational data. **Response:** Thank you for your valuable feedback regarding our manuscript. We appreciate your observation about the language used in relation to the pooled odds ratios. We have revised the interpretations throughout the manuscript to clearly reflect associations rather than causality, and we have made sure to acknowledge the limitations inherent in cross-sectional observational data. We believe these revisions strengthen our work, and we are grateful for your constructive feedback
6. **Comment:** The discussion could better contextualize findings within: Broader Sub-Saharan African data and differences in working conditions across regions of Ethiopia. Pls expand the discussion on contextual and structural factors affecting WMSDs among nurses in low-income settings

**Response:** We appreciate the reviewer’s insightful comment. In response, we have expanded the Discussion to incorporate broader Sub-Saharan African evidence, which situates our findings within the regional context and clarifies both similarities and inconsistencies across studies.

7. **Comment:** Language and grammar require minor revision throughout the manuscript.

**Response:** Thank you for your constructive feedback regarding the language and grammar in our manuscript. We have carefully reviewed the entire document and made the necessary minor revisions to enhance clarity and correctness.

8. **Comment:** Ensure consistent use of terms (e.g., WMSD vs. WMSDs).

**Response:** Thank you for your insightful comment regarding the consistency of term usage in our manuscript, specifically concerning the terms WMSD and WMSDs. We have addressed this issue by ensuring consistent terminology throughout the document.

9. **Comment:** Tables and figures should be checked for consistency in formatting and labeling.

**Response:** Thank you for your valuable feedback regarding the formatting and labeling of our tables and figures. We have thoroughly reviewed and corrected these elements to ensure consistency throughout the manuscript

10. **Comment:** Confidence interval formatting should be standardized.

**Response:** Thank you very much for your feedback regarding the standardization of confidence interval formatting. We have addressed this issue and ensured that the formatting is consistent throughout the manuscript. We appreciate your attention to detail, which has greatly improved our work.

11. **Comment:** The PRISMA flow diagram should strictly follow PRISMA 2020 terminology.

**Response:** Thank you for your valuable feedback regarding the PRISMA flow diagram. We have made the necessary revision of PRISMA flow diagram (Fig 1) to ensure that it strictly adheres to the PRISMA 2020 terminology. We appreciate your attention to detail, which has helped enhance the accuracy of our manuscript

12. **Comment:** Revise the abstract to:
    • Briefly mention the quality appraisal method
    • Rephrase novelty claims
    • Ensure associations are not interpreted causally

**Response:** Thank you for your valuable feedback. We have incorporated the quality appraisal method into the abstract and rephrased the novelty claims for clarity. Additionally, we have ensured that the associations are presented without implying causal interpretations.

13. **Comment:** Introduction
    • Strengthen the rationale for a country-specific meta-analysis.
    • Explicitly distinguish how this review adds to existing global or African reviews.

**Response:** Thank you for your insightful comments regarding the introduction of our manuscript. We have strengthened the rationale for conducting a country-specific meta-analysis and clearly distinguished how our review contributes to the existing global and African literature

14. **Comment:** Methods
    • Explicitly report any deviations from the registered protocol.
    • Provide full search strings for each database as supplementary material.
    • Clearly state language and date restrictions.
    • Describe how Google Scholar results were screened and limited.
    • Clarify how WMSDs were operationally defined and handled across studies.
    • Explore sources of heterogeneity where possible.
    • Clearly justify pooling of odds ratios from observational studies.

**Response:** Thank you for your detailed comments on the methods section. We appreciate your constructive feedback and have addressed each point raised. We have explicitly reported any deviations from the registered protocol, provided full search strings for each database as supplementary material, and clarified the language and date restrictions. Additionally, we have detailed the screening and limitations applied to Google Scholar results, clarified the operational definition and handling of WMSDs across studies, and explored potential sources of heterogeneity. Finally, we have justified the pooling of odds ratios from observational studies within the revised manuscript.

15. **Comment:** Results
    • Revise figures and tables for clarity and PRISMA compliance.

**Response:** Thank you for your feedback regarding the figures and tables in our manuscript. We have revised them for clarity and ensured that they comply with PRISMA guidelines. We appreciate your guidance, which has helped enhance the overall quality of our work

16. **Comment:** Discussion
    • Emphasize observational nature and avoid causal language.
    • Expand limitations section.
    • Discuss implications within healthcare system constraints.
    • Expand and deepen the limitations section.
    • Align conclusions more closely with the observational evidence.
    • PRISMA checklist should be submitted.
    • Data availability statement should clarify extracted data access.

**Response:** Thank you for your insightful feedback on the discussion section. We appreciate your recommendations and have addressed each point you raised. We have emphasized the observational nature of the study to avoid causal language, expanded the limitations section, and discussed implications within the constraints of the healthcare system. Additionally, we have aligned the conclusions more closely with the observational evidence, submitted the PRISMA checklist, and clarified the data availability statement regarding access to the extracted data in the revised manuscript

**Reviewer #3**

1. **Comment:** The background section provides a comprehensive and well-structured overview of work-related musculoskeletal disorders (WMSDs), clearly defining the concept and presenting its various synonymous terms. However, the background could be further strengthened by improving clarity and conciseness, as some sections contain repetitive information regarding prevalence and risk factors. In addition, although the authors state that no prior meta-analyses have been conducted on WMSDs among nurses in Ethiopia, this claim would benefit from a more explicit justification or citation to reinforce its validity. The final paragraph could more explicitly articulate how this study will advance existing knowledge, particularly in terms of informing policy, ergonomic interventions.

**Response:** Thank you for your insightful appreciation on the strength of our manuscript and comments regarding the background section on work-related musculoskeletal disorders (WMSDs). We have revised the text to enhance clarity and eliminate repetitive information about the prevalence and risk factors of WMSDs. This streamlining ensures a more focused presentation of the key points. To strengthen our assertion that no prior meta-analyses have been conducted on WMSDs among nurses in Ethiopia, we have included additional citations and justifications. This clarification will better support our claim and reinforce the significance of our research. In the final paragraph, we have explicitly articulated how this study will advance existing knowledge. We highlighted the potential impact on informing policy and developing effective ergonomic interventions designed for nurses, emphasizing the practical implications of our findings. We appreciate your guidance in enhancing the robustness of our work and believe these revisions will significantly improve the clarity and impact of our background section.

2. **Comment:** The methods section is clearly structured and transparent, registration of the study protocol in PROSPERO enhances the credibility and methodological rigor of the research, while adherence to the PRISMA 2020 guidelines further supports the study’s transparency and reproducibility. The methods would benefit from a more explicit presentation of complete search strings and whether reference list screening or manual searching was performed, which would strengthen reproducibility. The application of the PECOs framework for eligibility criteria is appropriate. However, the exclusion of gray literature beyond unpublished studies (e.g., theses or institutional reports) is not fully clarified, which may introduce publication bias. The use of the Joanna Briggs Institute (JBI) critical appraisal checklist for quality assessment is appropriate for observational studies. However, the critique notes that classifying all studies as low risk of bias may warrant further justification, as observational studies commonly exhibit at least moderate risk in certain domains. The statistical analysis section demonstrates a strong methodological foundation and appropriate use of advanced meta-analytic techniques. The use of STATA version 17 and application of a random-effects meta-analysis model (DerSimonian and Laird method) are appropriate.

**Response:** Thank you for your valuable feedback on our methods section. We appreciate your acknowledgment of the structured presentation and the registration of our study in PROSPERO, which indeed enhances the credibility of our research. We have submitted the complete search strings for each specific database as supplemental file and clarified on reference list screening and manual searching, additionally, we understand the importance of addressing the exclusion of gray literature; we provide a clearer rationale to minimize potential publication bias. Your comments on the use of the Joanna Briggs Institute critical appraisal checklist are also well-received, and we justify the classification of all studies as low risk of bias, recognizing the complexities present in observational studies. Thank you again for your insights.

3. **Comment:** The results section is clearly organized, logically sequenced, and consistent with PRISMA reporting standards. However, there appears to be a numerical inconsistency in the reporting of excluded studies (the number excluded at the title/abstract stage exceeds the number remaining after duplicate removal), which should be clarified to avoid confusion and ensure internal consistency.

**Response:** Thank you very much for your insightful comment regarding the results section. We appreciate your attention to detail and have corrected the numerical inconsistency you identified, which occurred due to a typographical error.

4. **Comment:** The characteristics of the included studies are presented comprehensively, with clear reporting of geographic distribution, study design, sample size, measurement tools, and prevalence estimates. However, notable variation in measurement tools (NMQ, DMQ, SSQ, VAS) introduces methodological heterogeneity that could affect prevalence estimates and should be more explicitly acknowledged as a limitation. The analysis of associated factors is a valuable addition to the study and enhances its practical relevance. The identification of sex, job stress, prolonged standing, and work experience as significant predictors aligns with existing occupational health literature. However, several concerns should be noted. First, the high heterogeneity observed in some pooled associations (e.g., prolonged standing and sleep disturbance) raises concerns about the reliability of these estimates. Pooling results with I² values exceeding 75% should be interpreted cautiously, and subgroup or sensitivity analyses specific to these variables would strengthen the findings. Second, the use of adjusted odds ratios pooled across studies with different covariate adjustments may compromise internal validity. Differences in confounding control across studies are not adequately addressed and may bias the pooled estimates.

**Response:** Thank you for your comprehensive feedback on the study's characteristics and analysis. We appreciate your recognition of the strengths in our reporting as well as your insightful observations regarding methodological heterogeneity. We have addressed the variation in measurement tools and explicitly acknowledged this as a limitation.

Regarding the high heterogeneity observed in pooled associations, we have taken your suggestion into account and included subgroup and sensitivity analyses specific to these variables in the revised manuscript. Additionally, we have clarified our approach to adjusted odds ratios and discussed the implications of different covariate adjustments across studies to enhance the internal validity of our findings. Thank you once again for your valuable input

5. **Comment:** The discussion section demonstrates a clear understanding of the study findings and effectively situates the results within the context of existing international and regional literature. The comparative analysis with findings from other countries and global systematic reviews is a major strength. However, the discussion would benefit from a deeper exploration of contextual factors, such as healthcare infrastructure, staffing ratios, ergonomic practices, and resource availability, which may explain observed differences in prevalence across countries. The association between job stress and WMSDs is well-articulated and supported by international evidence. The authors provide a coherent explanation linking psychological stress to musculoskeletal symptoms through fatigue and pain perception mechanisms. However, the discussion would benefit from acknowledging the high heterogeneity observed in this association and cautioning readers about overgeneralization. Similarly, the interpretation of work experience as a risk factor is logical and consistent with existing studies. However, the discussion could further differentiate whether increased risk is due to cumulative exposure, aging, or prolonged exposure to poor ergonomic conditions, which are not mutually exclusive explanations.

**Response:** We thank the reviewer for the constructive feedback. In response, we have expanded the Discussion to include contextual factors to better explain differences in prevalence across countries. We have also acknowledged the substantial heterogeneity observed in the job stress association and cautioned against overgeneralization. Furthermore, we have clarified that the increased risk with longer work experience may reflect cumulative exposure, aging, and prolonged exposure to poor ergonomic conditions.

6. **Comment:** The strengths and limitations section is appropriately positioned and clearly articulated. However, the limitations section, while accurate, could be more comprehensive. The reliance on cross-sectional studies is appropriately acknowledged, as it limits causal inference. Additional limitations that warrant discussion include:

1- Reliance on self-reported measures, which may introduce recall and reporting bias.

2- Variability in measurement tools used across studies, potentially affecting prevalence comparability.

3- Presence of publication bias, which, although addressed statistically, may still influence pooled estimates.

4- Limited representation from certain Ethiopian regions, which may affect national generalizability.

**Response:** Thank you very much for your thoughtful feedback on the strengths and limitations section. We appreciate your suggestion for a more comprehensive discussion of the limitations.

In response, we have expanded the limitations section to include additional factors such as the reliance on self-reported measures, which may introduce recall and reporting bias, and the presence of publication bias. We also acknowledge the limited representation from certain Ethiopian regions, which could affect the generalizability of our findings.

To address the variability in measurement tools used across studies, we have conducted a subgroup analysis based on these tools to ensure robustness in our findings.

Thank you again for your valuable insights, which have greatly improved the clarity of our manuscript.

7. **Comment:** The conclusions are concise, relevant, and directly aligned with the study objectives. However, the conclusion section appears incomplete, ending abruptly before fully summarizing all identified associated factors. Additionally, the conclusions could be strengthened by offering specific recommendations, such as ergonomic training, workplace redesign, and policy-level interventions tailored to the nursing workforce. Also, the conclusions would benefit from a brief mention of future research directions, such as the need for longitudinal studies, interventional trials, or qualitative research.

**Response:** Thank you very much for your insightful comments regarding the conclusions section. We appreciate your suggestion to enhance its completeness and relevance. In response, we have added specific recommendations, such as ergonomic training, and policy-level interventions tailored to the nursing workforce. Additionally, we now include future research directions, emphasizing the need for qualitative research and longitudinal studies or interventional trials.
