# Supplementary material for: A systematic review and meta-analysis on the epidemiology of work-related musculoskeletal disorders among nurses in Ethiopia
Source: PLoS One. 2026 Jul 22;21(7):e0354484. doi: 10.1371/journal.pone.0354484 (PMC13390853; doi:10.1371/journal.pone.0354484)
Supplement: Attachment — Submitted filename: New Microsoft Word Document (2).docx [file pone.0354484.s006.docx]

**Manuscript ID:** PONE-D-25-47479R1
**Title:** *A systematic review and meta-analysis on the epidemiology of work-related musculoskeletal disorders among nurses in Ethiopia*

Overall Evaluation

The authors have made substantial and sincere efforts to address the reviewers’ comments. The revised manuscript demonstrates marked improvement in transparency, methodological reporting, and discussion depth. Many of the major concerns raised in the initial review particularly those related to PRISMA adherence, search strategy reporting, subgroup analyses, and contextual discussion—have now been addressed.

However, several important issues remain, mainly related to interpretation of heterogeneity, pooling of adjusted odds ratios, and internal consistency. These issues do not invalidate the study but should be addressed before final acceptance.

Major Comments

1. PRISMA Compliance and Reporting Transparency — *Largely Resolved*

✔ The authors now explicitly state adherence to PRISMA 2020, provide a flow diagram, and submit a PRISMA checklist.
✔ Search dates, databases, and inclusion criteria are clearer.
✔ Supplementary materials now include detailed search strategies.

Remaining issue:

- Although PRISMA compliance is stated, the manuscript would benefit from a brief explicit statement confirming that all PRISMA 2020 items were met, with reference to the submitted checklist.

Recommendation: Minor clarification only.

2. Search Strategy and Reproducibility — *Mostly Resolved*

✔ Full electronic search strategies are provided as supplementary material.
✔ Language and date restrictions are now explicitly stated.
✔ Justification for Google Scholar use is included.

Remaining issue:

- The authors explicitly state that reference list screening and manual searching were not performed. While acceptable, this should be acknowledged as a limitation, as it may affect completeness.

Recommendation: Add one sentence in the limitations section.

3. Study Quality Assessment and Risk of Bias — *Partially Resolved*

✔ Use of the JBI critical appraisal checklist is appropriate and clearly described.
✔ Independent assessment by multiple reviewers is reported.

⚠ Concern remains:

- All included studies are classified as “low risk of bias,” which is unusual for cross-sectional observational studies.
- The justification provided is brief and may not fully convince readers.

Recommendation:

- Temper the language to acknowledge that “low risk” reflects methodological adequacy within JBI criteria, but residual bias typical of cross-sectional designs cannot be excluded.

4. Heterogeneity and Subgroup Analyses — *Improved but Interpretation Needs Caution*

✔ Subgroup analyses by region, year, sample size, body areas, and measurement tool have been added and strengthen the analysis.
✔ Sensitivity analyses are reported.

⚠ Critical issue:

- Some pooled associations (e.g., prolonged standing, sleep disturbance) show very high heterogeneity (I² > 85–90%), yet are still presented as significant findings.

Recommendation:

- Interpret these pooled estimates more cautiously and explicitly state that high heterogeneity limits confidence in these specific associations.
- Avoid emphasizing these findings in the conclusion.

5. Pooling of Adjusted Odds Ratios — *Still a Methodological Weakness*

⚠ The manuscript pools adjusted odds ratios from studies with different covariate sets, which may compromise internal validity.

Although the authors now acknowledge this issue, the limitation remains methodologically important.

Recommendation:

- Explicitly state that pooled AORs should be interpreted as approximate summaries, not precise effect estimates.
- Consider adding a brief justification for why meta-regression was not feasible (e.g., limited number of studies per factor).

6. Language Suggesting Causality — *Mostly Resolved*

✔ The authors have revised much of the language to reflect associations rather than causation.
✔ The observational nature of the evidence is now acknowledged.

Minor remaining issue:

- A few phrases in the Discussion and Conclusion still imply risk reduction or prevention effects.

Recommendation: Final language polishing to ensure consistent non-causal framing.

Minor Comments

1. Internal consistency:
   - Double-check numerical consistency between the PRISMA flow diagram and the Results text (earlier inconsistencies were corrected, but a final check is advised).
2. Terminology:
   - Consistent use of *WMSDs* is improved; continue to ensure uniform usage across figures and tables.
3. Tables:
   - Table 2 would benefit from a footnote reminding readers that pooled AORs are derived from heterogeneous adjustment models.
