# Supplementary material for: A systematic review and meta-analysis on the epidemiology of work-related musculoskeletal disorders among nurses in Ethiopia
Source: PLoS One. 2026 Jul 22;21(7):e0354484. doi: 10.1371/journal.pone.0354484 (PMC13390853; doi:10.1371/journal.pone.0354484)
Supplement: Attachment — Submitted filename: Response_to_Reviewers_auresp_2.docx [file pone.0354484.s009.docx]

**Editor’s comment**

1. **Comment:** If the reviewer comments include a recommendation to cite specific previously published works, please review and evaluate these publications to determine whether they are relevant and should be cited. There is no requirement to cite these works unless the editor has indicated otherwise.

**Response:** Thank you for your guidance. We appreciate your emphasis on the importance of relevant citations. However, we would like to clarify that the reviewer did not recommend any specific previously published works for us to cite

2. **Comment:** Please review your reference list to ensure that it is complete and correct. If you have cited papers that have been retracted, please include the rationale for doing so in the manuscript text, or remove these references and replace them with relevant current references. Any changes to the reference list should be mentioned in the rebuttal letter that accompanies your revised manuscript. If you need to cite a retracted article, indicate the article’s retracted status in the References list and also include a citation and full reference for the retraction notice.

**Response:** Thank you for this request. We reviewed the reference list and confirm that it is complete and correct. In addition, no retracted papers are cited in our manuscript; therefore no changes were required.

**Review Comments to the Author**

**Reviewer #2**

Overall Evaluation

1. **Comment**: The authors have made substantial and sincere efforts to address the reviewers’ comments. The revised manuscript demonstrates marked improvement in transparency, methodological reporting, and discussion depth. Many of the major concerns raised in the initial review particularly those related to PRISMA adherence, search strategy reporting, subgroup analyses, and contextual discussion—have now been addressed.

However, several important issues remain, mainly related to interpretation of heterogeneity, pooling of adjusted odds ratios, and internal consistency. These issues do not invalidate the study but should be addressed before final acceptance.

**Response:** Thank you for your careful review and encouraging comments. We appreciate that you recognize the improvements in transparency, methods reporting, and discussion. We have carefully considered the remaining issues you raised—interpretation of heterogeneity, pooling of adjusted odds ratios, and internal consistency—and have addressed them in the revised manuscript.

Major comments

2. **Comment:** PRISMA Compliance and Reporting Transparency — Largely Resolved

✔ The authors now explicitly state adherence to PRISMA 2020, provide a flow diagram, and submit a PRISMA checklist.

✔ Search dates, databases, and inclusion criteria are clearer.

✔ Supplementary materials now include detailed search strategies.

Remaining issue:

• Although PRISMA compliance is stated, the manuscript would benefit from a brief explicit statement confirming that all PRISMA 2020 items were met, with reference to the submitted checklist.

Recommendation: Minor clarification only

**Response**: Thank you for your confirmation and constructive feedback.

We have added a brief, explicit statement to the revised manuscript clarifying PRISMA 2020 compliance.

3. **Comment:** Search Strategy and Reproducibility — Mostly Resolved

✔ Full electronic search strategies are provided as supplementary material.

✔ Language and date restrictions are now explicitly stated.

✔ Justification for Google Scholar use is included.

Remaining issue:

• The authors explicitly state that reference list screening and manual searching were not performed. While acceptable, this should be acknowledged as a limitation, as it may affect completeness. Recommendation: Add one sentence in the limitations section.

**Response:** Thank you for your confirmation and constructive feedback. We have addressed the remaining comment by adding the following sentence to the Limitations section: "we did not perform reference-list screening or manual searches of identified articles; this may have reduced the completeness of study identification" Thank you once again for your helpful suggestions

4. **Comment:** Study Quality Assessment and Risk of Bias — Partially Resolved

✔ Use of the JBI critical appraisal checklist is appropriate and clearly described.

✔ Independent assessment by multiple reviewers is reported.

⚠ Concern remains:

• All included studies are classified as “low risk of bias,” which is unusual for cross-sectional observational studies.

• The justification provided is brief and may not fully convince readers.

Recommendation:

• Temper the language to acknowledge that “low risk” reflects methodological adequacy within JBI criteria, but residual bias typical of cross-sectional designs cannot be excluded.

**Response:** Thank you very much for this helpful observation. We have addressed the concern in the revised manuscript under “Quality assessment and risk of bias” section.

5. **Comment:** Heterogeneity and Subgroup Analyses — Improved but Interpretation Needs Caution

✔ Subgroup analyses by region, year, sample size, body areas, and measurement tool have been added and strengthen the analysis.

✔ Sensitivity analyses are reported.

⚠ Critical issue:

• Some pooled associations (e.g., prolonged standing, sleep disturbance) show very high heterogeneity (I² > 85–90%), yet are still presented as significant findings.

Recommendation:

• Interpret these pooled estimates more cautiously and explicitly state that high heterogeneity limits confidence in these specific associations.

• Avoid emphasizing these findings in the conclusion

**Response:** Thank you for this important guidance. In the previously submitted manuscript we explicitly stated that high heterogeneity limits confidence in these specific associations (lines 340–343, 352–354 etc). We have tempered the interpretation of these findings throughout the Discussion to reflect this uncertainty. However, we note that outright removal of these results from the Conclusion is not scientifically warranted, because the pooled estimates—despite high heterogeneity—still provide useful evidence that we report transparently alongside caveats about their limited confidence. We therefore retained the findings in the Conclusion. Thank you once again for your helpful suggestions.

6. **Comment:** Pooling of Adjusted Odds Ratios — Still a Methodological Weakness

⚠ The manuscript pools adjusted odds ratios from studies with different covariate sets, which may compromise internal validity.

Although the authors now acknowledge this issue, the limitation remains methodologically important.

Recommendation:

• Explicitly state that pooled AORs should be interpreted as approximate summaries, not precise effect estimates.

• Consider adding a brief justification for why meta-regression was not feasible (e.g., limited number of studies per factor)

**Response:** Thank you for this important methodological point. We have clarified in the revised manuscript under statistical analysis section that pooled adjusted odds ratios (PAORs) are presented as approximate summary measures—not precise effect estimates—because included studies adjusted for different covariate sets. We also explain in the Strengths and Limitations section that meta‑regression was not feasible due to the limited number of studies reporting comparable adjusted estimates per exposure

7. **Comment:** Language Suggesting Causality — Mostly Resolved

✔ The authors have revised much of the language to reflect associations rather than causation.

✔ The observational nature of the evidence is now acknowledged.

Minor remaining issue:

• A few phrases in the Discussion and Conclusion still imply risk reduction or prevention effects.

Recommendation: Final language polishing to ensure consistent non-causal framing.

**Response:** Thank you for this helpful suggestion. We have completed a final language polish to remove causal wording and ensure consistent non‑causal framing throughout the Discussion and Conclusion.

Minor comments

8. **Comment:** Internal consistency:

o Double-check numerical consistency between the PRISMA flow diagram and the Results text (earlier inconsistencies were corrected, but a final check is advised)

**Response:** Thank you for this careful check. We have performed a final verification and confirm that numerical counts in the PRISMA flow diagram and the Results text are now consistent

9. **Comment:** Terminology:

o Consistent use of WMSDs is improved; continue to ensure uniform usage across figures and tables

**Response:** Thank you. We have standardized the terminology throughout the manuscript and confirm that 'WMSDs' is used uniformly across the text, figures, and tables.

10. **Comment:** Tables:

o Table 2 would benefit from a footnote reminding readers that pooled AORs are derived from heterogeneous adjustment models

**Response:** Thank you. We have added a statement below Table 2 noting that PAOR should be interpreted as approximate summaries, rather than precise effect estimates due to substantial variability of covariates observed across studies.

**Reviewer #3**

1. **Comment:** Regarding the introduction, strong epidemiological justification, clear research gap, comprehensive risk factor coverage, and strong contextual relevance. If possible, reduce the Redundancy.

Protocol registration, PRISMA adherence, rigorous statistical methods, dual-review processes are major strengths. Kindly, describe the methods for pooling adjusted odds ratios (e.g., log transformation, heterogeneity handling, model selection).

**Response:** Thank you very much for these positive and constructive comments. We appreciate your recognition of the manuscript’s epidemiological justification, clear research gap, comprehensive risk‑factor coverage, and contextual relevance, as well as the strengths you noted (protocol registration, PRISMA adherence, rigorous statistical methods, and dual independent review).

Regarding the request to describe methods for pooling adjusted odds ratios, we briefly described this in the Methods under the Statistical analysis subsection. Thank you once again — we believe your input has substantially improved the manuscript.
