# Supplementary material for: A systematic review and meta-analysis on the epidemiology of work-related musculoskeletal disorders among nurses in Ethiopia
Source: PLoS One. 2026 Jul 22;21(7):e0354484. doi: 10.1371/journal.pone.0354484 (PMC13390853; doi:10.1371/journal.pone.0354484)
Supplement: Attachment — Submitted filename: New Microsoft Word Document (5).docx [file pone.0354484.s008.docx]

Overall assessment

The paper addresses an important occupational health issue and has several strengths: protocol registration, PRISMA-based reporting, multiple database searching, duplicate screening/quality appraisal, subgroup analysis, publication-bias assessment, and sensitivity analysis. The study also provides a useful pooled estimate for WMSDs among nurses in Ethiopia.

That said, the manuscript still has problems in four main areas:

- English language and grammar
- Overstatement of findings in some places
- Methodological inconsistency in interpretation of pooled adjusted odds ratios
- Need for tighter scientific writing, especially in Abstract, Discussion, and Conclusion

**Major issues**

1. Language needs substantial polishing

The manuscript still contains many grammatical and style problems that reduce readability and credibility.

Examples:

“no comprehensive meta-analysis have been done” should be “has been done.”

“Joana Brigg Institute” should be “Joanna Briggs Institute.”

“≥ 5 year of work experience of” is grammatically incorrect.

“The protocol … has been registered … codeCRD420251136383.We didn’t make an amendment…” is awkward and not journal-ready.

“two researchers independently evaluate” should be past tense.

This is not just cosmetic. The paper needs a full professional English edit.

2. The conclusion still overreaches

The paper repeatedly acknowledges that pooled AORs are only approximate summaries because studies adjusted for different covariates, and that some associations had high heterogeneity.

However, the conclusion still moves too far toward intervention language:

“Reducing job stress and prolonged standing…” in the abstract conclusion

“it may be important to improve staffing ratios…” in the final conclusion

For a review based entirely on observational studies, the wording should stay with:

“were associated with”

“may warrant attention”

“should be interpreted cautiously”

“may inform occupational health planning”

Not with implied preventive effect unless clearly framed as a recommendation rather than evidence of effect.

3. High-heterogeneity associations are still emphasized too strongly

The manuscript appropriately notes high heterogeneity for job stress and especially prolonged standing.

But these factors are still emphasized in the abstract and conclusion almost as if they were robust effect estimates. That is too strong, especially for:

Prolonged standing: I² = 91.4%

Job stress: I² = 70.8%

These should remain in the results, but in the abstract and conclusion the authors should make clear that these associations were observed with substantial between-study variability.

4. The Discussion needs more scientific restraint Some explanatory statements are too assertive or simplistic.

For example, the sex explanation relies heavily on biological reasoning and muscle mass/testosterone differences.

That is possible, but it reads as overly deterministic. The discussion should also acknowledge:

- role differences and task allocation
- domestic workload
- ergonomics
- reporting differences
- workplace context

A more balanced interpretation would be stronger scientifically.

5. Some claims in the Introduction are overstated or awkwardly phrased

Examples:

- “This variability and scarcity of clear epidemiological understanding deterred the design of diagnostic criteria, the establishment of specialized services, and recruitment in intervention trials.”

This feels exaggerated and probably unsupported.

- “This study is a reliable, evidence-based basis…” should be rewritten more modestly.

The introduction should be tighter and more evidence-led.

**Methodological concerns**

6) Pooling adjusted odds ratios remains a limitation The authors now acknowledge that PAORs are approximate because covariates differed across studies. That is good.

Still, the manuscript should be even clearer that:

- pooled adjusted effects across heterogeneous models are not equivalent to pooled effects from harmonized adjustment sets
- this reduces comparability and internal validity
- these results are exploratory summaries, not precise quantitative causal estimates

This caution should appear not only in Methods and Table 2 footnote, but also briefly in the Abstract or Conclusion.

7) Some factor interpretations are statistically awkward For work experience:
- PAOR = 1.58 (1.08–2.31), I² = 38.6%, P = 0.196

This appears to mix the heterogeneity p-value with inferential interpretation. The result is statistically significant based on the confidence interval excluding 1, but the narrative should avoid confusing readers by presenting the heterogeneity p-value next to the effect without clarification.

Also, in Table 2 and surrounding text, formatting and presentation should be standardized to avoid ambiguity.

**Internal consistency and reporting**

8) Scope of outcome needs clearer wording

The paper is framed as WMSDs among nurses, but several included studies appear focused on single body regions, especially low back pain or ankle-foot pain.

The authors partly address this by subgrouping according to number of body areas assessed.

Still, they should state more explicitly that the pooled estimate combines studies with different anatomical scopes, which may affect comparability.

9. Reference and citation quality should be rechecked A few citations look weak or mismatched:

Reference 30 is a study among bank workers in Ethiopia, yet it is cited for work-related musculoskeletal disorder definition/associated factors in places where a more direct occupational health or nursing source would be stronger.

Some supporting statements in the discussion would benefit from more directly relevant sources and less generic explanation.

**Minor issues**

10)) Title and abstract wording

The title is acceptable, but the abstract needs line editing for grammar and flow.

For example:

- “Result” should be “Results”
- “its associated factors” should be “associated factors”
- avoid recommendation-style language in the conclusion

11. Tables need cleanup

Table 1 and Table 2 are useful, but formatting is rough and should be standardized for journal submission.

Examples:

- inconsistent spacing
- awkward variable labels
- inconsistent capitalization
- some row labels are not reader-friendly

12. Strengths and limitations

section should be rewritten The section is useful, but the language is weak and repetitive.

For instance:

- “intensifies transparency” is not natural scientific English
- “negate WMSDs” in the conclusion is incorrect usage
