# Supplementary material for: A systematic review and meta-analysis on the epidemiology of work-related musculoskeletal disorders among nurses in Ethiopia
Source: PLoS One. 2026 Jul 22;21(7):e0354484. doi: 10.1371/journal.pone.0354484 (PMC13390853; doi:10.1371/journal.pone.0354484)
Supplement: Attachment — Submitted filename: Response_to_Reviewers_auresp_3.docx [file pone.0354484.s010.docx]

**Editor’s Comment**

1. **Comment**: If the reviewer comments include a recommendation to cite specific previously published works, please review and evaluate these publications to determine whether they are relevant and should be cited. There is no requirement to cite these works unless the editor has indicated otherwise.

**Response**: Thank you for your guidance. We appreciate your emphasis on the importance of relevant citations. However, we would like to clarify that the reviewer did not recommend any specific previously published works for us to cite

2. **Comment**: Please review your reference list to ensure that it is complete and correct. If you have cited papers that have been retracted, please include the rationale for doing so in the manuscript text, or remove these references and replace them with relevant current references. Any changes to the reference list should be mentioned in the rebuttal letter that accompanies your revised manuscript. If you need to cite a retracted article, indicate the article’s retracted status in the References list and also include a citation and full reference for the retraction notice.

**Response**: Thank you for this request. We reviewed the reference list and confirm that it is complete and correct. In addition, no retracted papers are cited in our manuscript; therefore no changes were required.

**Review Comments**

**Overall assessment**

1. **Comment:** The paper addresses an important occupational health issue and has several strengths: protocol registration, PRISMA-based reporting, multiple database searching, duplicate screening/quality appraisal, subgroup analysis, publication-bias assessment, and sensitivity analysis. The study also provides a useful pooled estimate for WMSDs among nurses in Ethiopia. That said, the manuscript still has problems in four main areas: • English language and grammar

• Overstatement of findings in some places

• Methodological inconsistency in interpretation of pooled adjusted odds ratios

• Need for tighter scientific writing, especially in Abstract, Discussion, and Conclusion

**Response**: Thank you for the helpful feedback and for recognizing the strengths of our manuscript. We agree that the issues you identified—English language and grammar, overstatement of findings, inconsistencies in the interpretation of the pooled adjusted odds ratios, and the need for tighter scientific writing—require improvement. We have addressed each of these concerns in the revised manuscript, including improving the overall writing quality (particularly in the Abstract, Discussion, and Conclusion), carefully revising the interpretation and reporting of pooled adjusted odds ratios to ensure methodological consistency, and revising statements where the strength of conclusions may have been overstated.

**# Major issues**

2. **Comment**: Language needs substantial polishing

The manuscript still contains many grammatical and style problems that reduce readability and credibility.

Examples:

“No comprehensive meta-analysis have been done” should be “has been done.” “Joana Brigg Institute” should be “Joanna Briggs Institute.”

“≥ 5 year of work experience of” is grammatically incorrect.

“The protocol … has been registered … codeCRD420251136383.We didn’t make an amendment…” is awkward and not journal-ready.

“Two researchers independently evaluate” should be past tense.

This is not just cosmetic. The paper needs a full professional English edit.

**Response**: Thank you for your comment and for the specific examples. We agree that the manuscript requires substantial language polishing to improve readability and enhance its credibility. In the revised manuscript, we have corrected the grammatical and stylistic issues you highlighted (e.g., subject–verb agreement, proper institute naming, correct preposition and wording, and improved phrasing for protocol registration and amendment statements). We have also revised the relevant sentences to ensure consistent tense throughout the Methods section and to make the manuscript fully journal-ready.

3. **Comment**: The conclusion still overreaches

The paper repeatedly acknowledges that pooled AORs are only approximate summaries because studies adjusted for different covariates, and that some associations had high heterogeneity. However, the conclusion still moves too far toward intervention language:

“Reducing job stress and prolonged standing…” in the abstract conclusion

“it may be important to improve staffing ratios…” in the final conclusion

For a review based entirely on observational studies, the wording should stay with: “were associated with”

“may warrant attention”

“should be interpreted cautiously”

“may inform occupational health planning”

Not with implied preventive effect unless clearly framed as a recommendation rather than evidence of effect.

**Response**: Thank you for this important comment. We agree that, because the evidence is based entirely on observational studies, the conclusion should not imply preventive or causal effects and should remain focused on associations. In the revised manuscript, we have revised the Abstract and final Conclusion to replace intervention/causal language with appropriate association-based wording (e.g., using phrasing such as “were associated with,” and “may warrant attention”.

4. **Comment**: High-heterogeneity associations are still emphasized too strongly. The manuscript appropriately notes high heterogeneity for job stress and especially prolonged standing. But these factors are still emphasized in the abstract and conclusion almost as if they were robust effect estimates. That is too strong, especially for:

Prolonged standing: I² = 91.4%

Job stress: I² = 70.8%

These should remain in the results, but in the abstract and conclusion the authors should make clear that these associations were observed with substantial between-study variability.

**Response**: Thank you for this valuable comment. We agree that the abstract and conclusion currently emphasize job stress and prolonged standing too strongly relative to the substantial between-study heterogeneity observed. In the revised manuscript, we have revised the Abstract and final Conclusion to ensure that the pooled findings for prolonged standing and job stress are presented as associations with substantial variability across studies.

5. **Comment**: The Discussion needs more scientific restraint Some explanatory statements are too assertive or simplistic.

For example, the sex explanation relies heavily on biological reasoning and muscle mass/testosterone differences.

That is possible, but it reads as overly deterministic. The discussion should also acknowledge:

• role differences and task allocation

• domestic workload

• ergonomics

• reporting differences

• workplace context

A more balanced interpretation would be stronger scientifically

**Response**: Thank you for this valuable comment. In the revised manuscript, we have strengthened the Discussion by providing a more balanced, scientifically restrained interpretation. Specifically, we now acknowledge that the observed sex differences may reflect a combination of factors, including domestic workload

6. **Comment**: Some claims in the Introduction are overstated or awkwardly phrased

Examples:

• “This variability and scarcity of clear epidemiological understanding deterred the design of diagnostic criteria, the establishment of specialized services, and recruitment in intervention trials.”

This feels exaggerated and probably unsupported.

• “This study is a reliable, evidence-based basis…” should be rewritten more modestly.

The introduction should be tighter and more evidence-led.

**Response**: Thank you for this constructive comment. We agree that some statements in the introduction were overstated and that the wording should be more modest and evidence-led. In the revised manuscript, we have removed or reframed the exaggerated claims

7. **Comment**: Methodological concerns

Pooling adjusted odds ratios remains a limitation The authors now acknowledge that PAORs are approximate because covariates differed across studies. That is good.

Still, the manuscript should be even clearer that:

• pooled adjusted effects across heterogeneous models are not equivalent to pooled effects from harmonized adjustment sets

• this reduces comparability and internal validity

• these results are exploratory summaries, not precise quantitative causal estimates

This caution should appear not only in Methods and Table 2 footnote, but also briefly in the Abstract or Conclusion.

**Response**: Thank you for the comment. We agree that the limitation of pooling adjusted odds ratios requires clear emphasis, not only in the Methods and Table 2 footnote but also in the Conclusion. We therefore revised the Conclusion to explicitly state that the pooled adjusted effects are based on study-specific adjustment sets, which varied across included studies; hence, these pooled estimates are approximate exploratory summaries rather than harmonized-adjustment effects or precise quantitative causal estimates. This wording also clarifies that differences in covariates may reduce comparability and internal validity across studies.

8. **Comment**: Some factor interpretations are statistically awkward For work experience:

• PAOR = 1.58 (1.08–2.31), I² = 38.6%, P = 0.196

This appears to mix the heterogeneity p-value with inferential interpretation. The result is statistically significant based on the confidence interval excluding 1, but the narrative should avoid confusing readers by presenting the heterogeneity p-value next to the effect without clarification.

Also, in Table 2 and surrounding text, formatting and presentation should be standardized to avoid ambiguity.

**Response**: Thank you for the comment. We agree that the way the heterogeneity P-value was presented alongside the pooled adjusted odds ratio (AOR) could be confusing and mix different statistical interpretations. In the revised manuscript, we have removed the heterogeneity P-values from the surrounding text. We have also standardized the formatting and presentation across the relevant sections to improve clarity and avoid ambiguity.

9. **Comment**: Scope of outcome needs clearer wording

The paper is framed as WMSDs among nurses, but several included studies appear focused on single body regions, especially low back pain or ankle-foot pain.

The authors partly address this by subgrouping according to number of body areas assessed.

Still, they should state more explicitly that the pooled estimate combines studies with different anatomical scopes, which may affect comparability.

**Response**: Thank you for this helpful comment. In response, we have revised the manuscript by adding explicit wording in the Outcome measurement section to clarify that the included studies differed in anatomical scope (single-region outcomes such as low back pain or ankle/foot pain versus multi-region assessments). We also emphasize that the pooled prevalence estimate therefore combines studies with heterogeneous anatomical definitions, which may affect comparability, and we explain that subgroup analyses were conducted based on the number of body regions assessed to partially address this heterogeneity.

10. **Comment**: Reference and citation quality should be rechecked A few citations look weak or mismatched: Reference 30 is a study among bank workers in Ethiopia, yet it is cited for work-related musculoskeletal disorder definition/associated factors in places where a more direct occupational health or nursing source would be stronger.

Some supporting statements in the discussion would benefit from more directly relevant sources and less generic explanation

**Response**: Thank you for this comment. In the revised manuscript, we rechecked the references and also added additional references.

**# Minor issues**

11. **Comment**: Title and abstract wording

The title is acceptable, but the abstract needs line editing for grammar and flow.

For example:

• “Result” should be “Results”

• “its associated factors” should be “associated factors”

• avoid recommendation-style language in the conclusion

**Response**: Thank you for the comment. We agree that the abstract requires line editing for grammar, clarity, and flow. In the revised manuscript, we have corrected the identified issues. We have also removed any recommendation-style language in the conclusion and should be interpreted as associations with possible between-study variability.

12. **Comment**: Tables need cleanup

Table 1 and Table 2 are useful, but formatting is rough and should be standardized for journal submission.

Examples:

• inconsistent spacing

• awkward variable labels

• inconsistent capitalization

• some row labels are not reader-friendly

**Response**: Thank you for this comment. We have reviewed Tables 1 and 2 in the revised manuscript and improved their formatting to meet journal submission standards.

13. **Comment**: Strengths and limitations

section should be rewritten The section is useful, but the language is weak and repetitive.

For instance:

• “intensifies transparency” is not natural scientific English

• “negate WMSDs” in the conclusion is incorrect usage

**Response**: Thank you for this comment. In the revised manuscript, we rewrote the section to improve academic language quality and reduce repetition.
