# Supplementary material for: A systematic review and meta-analysis on the epidemiology of work-related musculoskeletal disorders among nurses in Ethiopia
Source: PLoS One. 2026 Jul 22;21(7):e0354484. doi: 10.1371/journal.pone.0354484 (PMC13390853; doi:10.1371/journal.pone.0354484)
Supplement: Peer review history

# Peer review: Decision Letter 0

**DOI:** 10.1371/journal.pone.0354484.r001

**Authors:** Sohel Ahmed

29 Dec 2025

PONE-D-25-47479

A systematic review and meta-analysis on the epidemiology of work-related musculoskeletal disorders among nurses in Ethiopia

PLOS One

Dear Dr. Demeke,

Thank you for submitting your manuscript to PLOS ONE. After careful consideration, we feel that it has merit but does not fully meet PLOS ONE’s publication criteria as it currently stands. Therefore, we invite you to submit a revised version of the manuscript that addresses the points raised during the review process.

Please submit your revised manuscript by Feb 12 2026 11:59PM. If you will need more time than this to complete your revisions, please reply to this message or contact the journal office at plosone@plos.org. When you're ready to submit your revision, log on to https://www.editorialmanager.com/pone/ and select the 'Submissions Needing Revision' folder to locate your manuscript file.

Please include the following items when submitting your revised manuscript:

- A letter that responds to each point raised by the academic editor and reviewer(s). You should upload this letter as a separate file labeled 'Response to Reviewers'.
- A marked-up copy of your manuscript that highlights changes made to the original version. You should upload this as a separate file labeled 'Revised Manuscript with Track Changes'.
- An unmarked version of your revised paper without tracked changes. You should upload this as a separate file labeled 'Manuscript'.

If you would like to make changes to your financial disclosure, please include your updated statement in your cover letter. Guidelines for resubmitting your figure files are available below the reviewer comments at the end of this letter.

If applicable, we recommend that you deposit your laboratory protocols in protocols.io to enhance the reproducibility of your results. Protocols.io assigns your protocol its own identifier (DOI) so that it can be cited independently in the future. For instructions see: https://journals.plos.org/plosone/s/submission-guidelines#loc-laboratory-protocols. Additionally, PLOS ONE offers an option for publishing peer-reviewed Lab Protocol articles, which describe protocols hosted on protocols.io. Read more information on sharing protocols at https://plos.org/protocols?utm_medium=editorial-email&utm_source=authorletters&utm_campaign=protocols.

We look forward to receiving your revised manuscript.

Kind regards,

Sohel Ahmed, MPT, MDMR

Academic Editor

PLOS One

Journal Requirements:

When submitting your revision, we need you to address these additional requirements.

1. Please ensure that your manuscript meets PLOS ONE's style requirements, including those for file naming. The PLOS ONE style templates can be found at

https://journals.plos.org/plosone/s/file?id=wjVg/PLOSOne_formatting_sample_main_body.pdf and

https://journals.plos.org/plosone/s/file?id=ba62/PLOSOne_formatting_sample_title_authors_affiliations.pdf

2. Please update your submission to use the PLOS LaTeX template. The template and more information on our requirements for LaTeX submissions can be found at http://journals.plos.org/plosone/s/latex.

3. Please upload a new copy of Figures 10 - 13 as the detail is not clear. Please follow the link for more information:  https://journals.plos.org/plosone/s/figures

4. Please include a new copy of Table S3 in your manuscript; the current table is difficult to read. Please follow the link for more information: https://journals.plos.org/plosone/s/tables

5. If the reviewer comments include a recommendation to cite specific previously published works, please review and evaluate these publications to determine whether they are relevant and should be cited. There is no requirement to cite these works unless the editor has indicated otherwise.

[Note: HTML markup is below. Please do not edit.]

Reviewers' comments:

Reviewer's Responses to Questions

**Comments to the Author**

1. Is the manuscript technically sound, and do the data support the conclusions?

The manuscript must describe a technically sound piece of scientific research with data that supports the conclusions. Experiments must have been conducted rigorously, with appropriate controls, replication, and sample sizes. The conclusions must be drawn appropriately based on the data presented.

Reviewer #1: Partly

Reviewer #2: Yes

Reviewer #3: Partly

**********

2. Has the statistical analysis been performed appropriately and rigorously?

Reviewer #1: I Don't Know

Reviewer #2: N/A

Reviewer #3: Yes

**********

3. Have the authors made all data underlying the findings in their manuscript fully available?

The PLOS Data policy requires authors to make all data underlying the findings described in their manuscript fully available without restriction, with rare exception (please refer to the Data Availability Statement in the manuscript PDF file). The data should be provided as part of the manuscript or its supporting information, or deposited to a public repository. For example, in addition to summary statistics, the data points behind means, medians and variance measures should be available. If there are restrictions on publicly sharing data—e.g. participant privacy or use of data from a third party—those must be specified.

Reviewer #1: No

Reviewer #2: Yes

Reviewer #3: Yes

**********

4. Is the manuscript presented in an intelligible fashion and written in standard English?

PLOS ONE does not copyedit accepted manuscripts, so the language in submitted articles must be clear, correct, and unambiguous. Any typographical or grammatical errors should be corrected at revision, so please note any specific errors here.

Reviewer #1: No

Reviewer #2: Yes

Reviewer #3: Yes

**********

5. Review Comments to the Author

Please use the space provided to explain your answers to the questions above. You may also include additional comments for the author, including concerns about dual publication, research ethics, or publication ethics. (Please upload your review as an attachment if it exceeds 20,000 characters)

Reviewer #1: Reviewer comment and suggestions

Title and Abstract

Strengths

• The objective is clear: estimating pooled prevalence and associated factors of WMSDs among nurses in Ethiopia.

• Key results (prevalence, associated factors) are concisely summarized.

Limitations

• The abstract may be overly long; it reads more like a truncated full manuscript than a standard abstract.

• Key elements like registration of the review (e.g., PROSPERO number) are missing.

• No statement about the certainty of evidence or grading (e.g., GRADE approach).

Introduction

Strengths

• Identifies a clear knowledge gap: lack of compiled meta-analytic evidence on WMSDs in Ethiopian nurses.

• Provides justification due to high vulnerability of nurses.

Limitations

• Insufficient context: Does not explain global WMSD prevalence or compare Ethiopia to other regions.

• Does not explicitly state a theoretical framework guiding which factors were expected to be associated.

Methods

Strengths

• Multiple reputable databases searched (PubMed, ScienceDirect, HINARI, Google Scholar).

• Inclusion of published and unpublished studies reduces publication bias.

• Use of standard analytical elements: random-effects model, heterogeneity assessment (I²), funnel plot, Egger’s test, trim-and-fill, sensitivity analysis.

Limitations and Concerns

a. No PRISMA Compliance Mentioned

The description should explicitly state adherence to PRISMA 2020 guidelines, including a flow diagram.

b. Search Strategy Not Fully Described

Missing:

o Search string(s)

o Language restrictions

o Search date ranges (only end date given)

o Screening process (number of reviewers, resolution of disagreements)

Without these details, replication is difficult.

c. Study Quality Assessment Not Reported

• No mention of quality or bias assessment tools (e.g., Newcastle–Ottawa Scale).

• The robustness of included studies cannot be evaluated.

d. Lack of Data Extracted Variables

The description does not specify:

o How WMSD was defined across studies

o Whether data extraction was done independently by two reviewers

o Adjusted vs. crude effect sizes

e. No Handling of Variation Between Studies

• WMSD definitions, measurement tools, and anatomical regions may differ widely.

• No subgroup analyses (e.g., by region, measurement tool, year of study), which could help explain heterogeneity.

Results

Strengths

• Provides pooled sample size and number of included studies.

• Reports pooled prevalence with confidence intervals.

• Identifies significant associated factors with pooled odds ratios.

Limitations

a. High Heterogeneity Likely

• Although I² is mentioned, its value is not reported.

• Prevalence studies typically show high heterogeneity; without subgroup/exploratory analyses, results may be unreliable.

b. No Visuals Reported

• Forest plot, funnel plot, trim-and-fill output, and PRISMA flow diagram are essential but not mentioned or described.

c. Limited Examination of Associated Factors

• Only four factors identified; unclear if others were tested (e.g., BMI, working hours, shift work, patient-handling practices).

d. No Sensitivity Analysis Results Reported

• Stated as conducted but results not summarized.

Discussion

Strengths

• Conclusion aligns with findings.

• Suggests practical implications (e.g., focus on reducing stress and standing time).

Limitations

• Does not compare pooled estimates with global or African data.

• Does not address:

o Potential reverse causation (cross-sectional designs dominate this field).

o Measurement bias (self-reported outcomes).

o Differences in workplace ergonomics across studies.

Conclusion

Strengths

• Acknowledges high prevalence and identifies actionable factors.

Limitations

• Recommendations are generic and lack specificity (e.g., ergonomic programs, workload redesign, staffing ratio improvement).

• Does not address research implications, such as the need for longitudinal or intervention studies.

Overall Strengths of the Study

• Addresses an important occupational health issue.

• Uses meta-analysis to synthesize evidence valuable for policymaking.

• Includes multiple databases and unpublished studies.

• Demonstrates methodological awareness (heterogeneity, publication bias).

Overall Limitations and Areas for Improvement

Methodological Limitations

• Insufficient reporting on search strategy and risk-of-bias assessment.

• Unclear adherence to PRISMA guidelines.

• No subgroup or meta-regression analyses.

• Unclear handling of inconsistent definitions of WMSD.

• High heterogeneity likely undermines pooled prevalence reliability.

Analytical Limitations

• Effect estimates may be confounded due to lack of adjusted ORs.

• No weighting or discussion of study quality.

Interpretation Limitations

• Overgeneralizes findings without accounting for workplace variability across Ethiopia.

Summary

The study provides useful pooled estimates of WMSDs among nurses in Ethiopia, showing a high prevalence (61.47%) and identifying significant associated factors. However, methodological weaknesses especially incomplete reporting of search strategy, lack of study quality assessment, unclear heterogeneity handling, and limited discussion of contextual factors reduce confidence in the pooled estimates. Strengthening transparency, performing subgroup analyses, and adhering strictly to PRISMA standards would significantly improve the study’s rigor and credibility.

Reviewer #2: For the authors:

• Although a PROSPERO registration number is provided, the manuscript does not clearly describe whether the review was conducted strictly according to the registered protocol. Pls. clarify deviations from the registered protocol and explicitly state adherence to the PROSPERO plan.

• The authors report searching multiple databases; however, the manuscript lacks: Full electronic search strategies, Search strings for each database, Justification for the use of Google Scholar and screening limits

pls. provide complete search strategies (preferably as supplementary material) to ensure reproducibility, as required by PRISMA 2020.

• While observational studies were included, the methodology does not sufficiently describe: the specific quality appraisal tool used and How risk of bias influenced interpretation of pooled results. Pls. clarify the tool used and discuss how study quality may have affected the meta-analysis findings.

• Substantial heterogeneity is expected in prevalence meta-analyses; however, the manuscript does not adequately explore potential sources of heterogeneity beyond reporting I². pls. consider additional subgroup or meta-regression analyses (e.g., by region, study setting, or assessment tool) or explicitly justify their absence.

• The manuscript reports pooled odds ratios for associated factors; however, the language occasionally implies causality. Pls. revise interpretations to clearly reflect associations, acknowledging the limitations of cross-sectional observational data.

• The discussion could better contextualize findings within: Broader Sub-Saharan African data and differences in working conditions across regions of Ethiopia. Pls expand the discussion on contextual and structural factors affecting WMSDs among nurses in low-income settings.

• Language and grammar require minor revision throughout the manuscript.

• Ensure consistent use of terms (e.g., WMSD vs. WMSDs).

• Tables and figures should be checked for consistency in formatting and labeling.

• Confidence interval formatting should be standardized.

• The PRISMA flow diagram should strictly follow PRISMA 2020 terminology.

Revise the abstract to:

• Briefly mention the quality appraisal method

• Rephrase novelty claims

• Ensure associations are not interpreted causally

Introduction

• Strengthen the rationale for a country-specific meta-analysis.

• Explicitly distinguish how this review adds to existing global or African reviews.

Methods

• Explicitly report any deviations from the registered protocol.

• Provide full search strings for each database as supplementary material.

• Clearly state language and date restrictions.

• Describe how Google Scholar results were screened and limited.

• Clarify how WMSDs were operationally defined and handled across studies.

• Explore sources of heterogeneity where possible.

• Clearly justify pooling of odds ratios from observational studies.

Results

• Revise figures and tables for clarity and PRISMA compliance.

Discussion

• Emphasize observational nature and avoid causal language.

• Expand limitations section.

• Discuss implications within healthcare system constraints.

• Expand and deepen the limitations section.

• Align conclusions more closely with the observational evidence.

• PRISMA checklist should be submitted.

• Data availability statement should clarify extracted data access.

Reviewer #3: The background section provides a comprehensive and well-structured overview of work-related musculoskeletal disorders (WMSDs), clearly defining the concept and presenting its various synonymous terms. However, the background could be further strengthened by improving clarity and conciseness, as some sections contain repetitive information regarding prevalence and risk factors. In addition, although the authors state that no prior meta-analyses have been conducted on WMSDs among nurses in Ethiopia, this claim would benefit from a more explicit justification or citation to reinforce its validity. The final paragraph could more explicitly articulate how this study will advance existing knowledge, particularly in terms of informing policy, ergonomic interventions.

The methods section is clearly structured and transparent, registration of the study protocol in PROSPERO enhances the credibility and methodological rigor of the research, while adherence to the PRISMA 2020 guidelines further supports the study’s transparency and reproducibility. The methods would benefit from a more explicit presentation of complete search strings and whether reference list screening or manual searching was performed, which would strengthen reproducibility. The application of the PECOs framework for eligibility criteria is appropriate. However, the exclusion of gray literature beyond unpublished studies (e.g., theses or institutional reports) is not fully clarified, which may introduce publication bias. The use of the Joanna Briggs Institute (JBI) critical appraisal checklist for quality assessment is appropriate for observational studies. However, the critique notes that classifying all studies as low risk of bias may warrant further justification, as observational studies commonly exhibit at least moderate risk in certain domains.

The statistical analysis section demonstrates a strong methodological foundation and appropriate use of advanced meta-analytic techniques. The use of STATA version 17 and application of a random-effects meta-analysis model (DerSimonian and Laird method) are appropriate.

The results section is clearly organized, logically sequenced, and consistent with PRISMA reporting standards. However, there appears to be a numerical inconsistency in the reporting of excluded studies (the number excluded at the title/abstract stage exceeds the number remaining after duplicate removal), which should be clarified to avoid confusion and ensure internal consistency. The characteristics of the included studies are presented comprehensively, with clear reporting of geographic distribution, study design, sample size, measurement tools, and prevalence estimates. However, notable variation in measurement tools (NMQ, DMQ, SSQ, VAS) introduces methodological heterogeneity that could affect prevalence estimates and should be more explicitly acknowledged as a limitation.

The analysis of associated factors is a valuable addition to the study and enhances its practical relevance. The identification of sex, job stress, prolonged standing, and work experience as significant predictors aligns with existing occupational health literature. However, several concerns should be noted. First, the high heterogeneity observed in some pooled associations (e.g., prolonged standing and sleep disturbance) raises concerns about the reliability of these estimates. Pooling results with I² values exceeding 75% should be interpreted cautiously, and subgroup or sensitivity analyses specific to these variables would strengthen the findings. Second, the use of adjusted odds ratios pooled across studies with different covariate adjustments may compromise internal validity. Differences in confounding control across studies are not adequately addressed and may bias the pooled estimates.

The discussion section demonstrates a clear understanding of the study findings and effectively situates the results within the context of existing international and regional literature.

The comparative analysis with findings from other countries and global systematic reviews is a major strength. However, the discussion would benefit from a deeper exploration of contextual factors, such as healthcare infrastructure, staffing ratios, ergonomic practices, and resource availability, which may explain observed differences in prevalence across countries.

The association between job stress and WMSDs is well-articulated and supported by international evidence. The authors provide a coherent explanation linking psychological stress to musculoskeletal symptoms through fatigue and pain perception mechanisms. However, the discussion would benefit from acknowledging the high heterogeneity observed in this association and cautioning readers about overgeneralization.

Similarly, the interpretation of work experience as a risk factor is logical and consistent with existing studies. However, the discussion could further differentiate whether increased risk is due to cumulative exposure, aging, or prolonged exposure to poor ergonomic conditions, which are not mutually exclusive explanations.

The strengths and limitations section is appropriately positioned and clearly articulated. However, the limitations section, while accurate, could be more comprehensive. The reliance on cross-sectional studies is appropriately acknowledged, as it limits causal inference. Additional limitations that warrant discussion include:

1- Reliance on self-reported measures, which may introduce recall and reporting bias.

2- Variability in measurement tools used across studies, potentially affecting prevalence comparability.

3- Presence of publication bias, which, although addressed statistically, may still influence pooled estimates.

4- Limited representation from certain Ethiopian regions, which may affect national generalizability.

The conclusions are concise, relevant, and directly aligned with the study objectives. However, the conclusion section appears incomplete, ending abruptly before fully summarizing all identified associated factors. Additionally, the conclusions could be strengthened by offering specific recommendations, such as ergonomic training, workplace redesign, and policy-level interventions tailored to the nursing workforce. Also, the conclusions would benefit from a brief mention of future research directions, such as the need for longitudinal studies, interventional trials, or qualitative research.

**********

6. PLOS authors have the option to publish the peer review history of their article (what does this mean?). If published, this will include your full peer review and any attached files.

If you choose “no”, your identity will remain anonymous but your review may still be made public.

**Do you want your identity to be public for this peer review?**  For information about this choice, including consent withdrawal, please see our Privacy Policy.

Reviewer #1: **Yes:**  REHEMA ABDALLAH

Reviewer #2: No

Reviewer #3: **Yes:**  Dr. Lareen Magdi El-Sayed Abo-Seif

**********

[NOTE: If reviewer comments were submitted as an attachment file, they will be attached to this email and accessible via the submission site. Please log into your account, locate the manuscript record, and check for the action link "View Attachments". If this link does not appear, there are no attachment files.]

To ensure your figures meet our technical requirements, please review our figure guidelines: https://journals.plos.org/plosone/s/figures

You may also use PLOS’s free figure tool, NAAS, to help you prepare publication quality figures: https://journals.plos.org/plosone/s/figures#loc-tools-for-figure-preparation.

NAAS will assess whether your figures meet our technical requirements by comparing each figure against our figure specifications.

# Author comment: Author response to Decision Letter 1

**DOI:** 10.1371/journal.pone.0354484.r002

8 Feb 2026

Editors Comment

1. Comment: Please ensure that your manuscript meets PLOS ONE's style requirements, including those for file naming. The PLOS ONE style templates can be found at https://journals.plos.org/plosone/s/file?id=wjVg/PLOSOne_formatting_sample_main_body.pdf and https://journals.plos.org/plosone/s/file?id=ba62/PLOSOne_formatting_sample_title_authors_affiliations.pdf

Response: Thank you for your feedback. We have thoroughly reviewed our manuscript and ensured that it meets all of PLOS ONE's style requirements, including those related to file naming conventions. We appreciate your guidance and have utilized the provided templates to align our submission accordingly.

2. Comment: Please update your submission to use the PLOS LaTeX template. The template and more information on our requirements for LaTeX submissions can be found at http://journals.plos.org/plosone/s/latex.

Response: Thank you for your suggestion. We have updated our submission to utilize the PLOS LaTeX template as per your requirements. We appreciate your guidance in ensuring our manuscript meets the necessary standards.

3. Comment: Please upload a new copy of Figures 10 - 13 as the detail is not clear. Please follow the link for more information: https://journals.plos.org/plosone/s/figures

Response: Thank you for your valuable feedback regarding Figures 10-13. We appreciate your input, as it greatly enhances the quality of our manuscript. We have uploaded a new copy of Figures 11-14, as the previous details were not clear. Please note that due to the addition of a new figure (Fig 7, subgroup analysis by measurement tool), the order of the figures has changed. Consequently, what were originally Figures 10-13 are now Figures 11-14.

4. Comment: Please include a new copy of Table S3 in your manuscript; the current table is difficult to read. Please follow the link for more information: https://journals.plos.org/plosone/s/tables

Response: Thank you for your feedback. We have revised Table S3 to enhance its readability by using shorter abbreviations for the questions and providing a clear description below the table. We appreciate your guidance in improving our manuscript.

5. Comment: If the reviewer comments include a recommendation to cite specific previously published works, please review and evaluate these publications to determine whether they are relevant and should be cited. There is no requirement to cite these works unless the editor has indicated otherwise.

Response: Thank you for your guidance. We appreciate your emphasis on the importance of relevant citations. However, we would like to clarify that the reviewer did not recommend any specific previously published works for us to cite.

Reviewers comment

Reviewer #1

1. Comment: Title and Abstract

Strengths

• The objective is clear: estimating pooled prevalence and associated factors of WMSDs among nurses in Ethiopia.

• Key results (prevalence, associated factors) are concisely summarized.

Limitations

• The abstract may be overly long; it reads more like a truncated full manuscript than a standard abstract.

• Key elements like registration of the review (e.g., PROSPERO number) are missing.

Response: Thank you for your positive feedback on the strengths of our manuscript. We have revised our abstract to better align with standard formatting. While the PROSPERO registration number was previously included at the end of the abstract, we have now moved it to the methods section for improved clarity and visibility. Additionally, we have included a statement regarding the assessment of the quality of the literature.

2. Comment: Introduction

Strengths

• Identifies a clear knowledge gap: lack of compiled meta-analytic evidence on WMSDs in Ethiopian nurses.

• Provides justification due to high vulnerability of nurses.

Limitations

• Insufficient context: Does not explain global WMSD prevalence or compare Ethiopia to other regions.

• Does not explicitly state a theoretical framework guiding which factors were expected to be associated.

Response: Thank you for your constructive feedback on the strengths of our manuscript. We have now included an explanation of the global prevalence of WMSDs and described the constraints faced in low- and middle-income countries, specifically in Ethiopia. Additionally, we have incorporated a theoretical framework (model) that explores the factors alongside those previously listed.

3. Comment: Methods

Strengths

• Multiple reputable databases searched (PubMed, ScienceDirect, HINARI, Google Scholar).

• Inclusion of published and unpublished studies reduces publication bias.

• Use of standard analytical elements: random-effects model, heterogeneity assessment (I²), funnel plot, Egger’s test, trim-and-fill, sensitivity analysis.

Response: Thank you for your thoughtful comments regarding the strengths of our manuscript! I truly appreciate your acknowledgment of the comprehensive methodology we've employed in our analysis.

Limitations and Concerns

a. No PRISMA Compliance Mentioned

The description should explicitly state adherence to PRISMA 2020 guidelines, including a flow diagram.

Response: Thank you for your valuable feedback regarding PRISMA compliance. While we previously mentioned it in the methods section, we appreciate your suggestion and have now explicitly stated our adherence to the PRISMA 2020 guidelines, including the flow diagram.

b. Search Strategy Not Fully Described

Missing:

o Search string(s)

o Language restrictions

o Search date ranges (only end date given)

o Screening process (number of reviewers, resolution of disagreements)

Without these details, replication is difficult.

Response: Thank you for your constructive feedback regarding the search strategy. We previously noted that no language restrictions or time bounds were applied in the inclusion criteria. For greater clarity, we have now relocated this information to the "Information Sources and Search Strategy" section. Additionally, we clarified that the studies included were from inception to August 14, 2025, with a specific search conducted from August 5 to August 14. Furthermore, the details of the search strategy can be found in Supplementary Table S2.

Regarding the screening process, we have outlined the number of reviewers and the resolution of disagreements in the "Study Selection Process" subsection. We appreciate your input, which has significantly improved the clarity of our manuscript.

c. Study Quality Assessment Not Reported

• No mention of quality or bias assessment tools (e.g., Newcastle–Ottawa Scale).

• The robustness of included studies cannot be evaluated.

Response: Thank you for your reminder regarding the quality assessment of the studies. However, we have clearly described this in the "Data Extraction, Study Quality, and Risk of Bias Assessment" subsection. Specifically, we assessed the methodological quality of the selected studies using the Joanna Briggs Institute (JBI) critical appraisal checklist. We appreciate your attention to this important aspect, as it ensures the robustness of our findings.

d. Lack of Data Extracted Variables

The description does not specify:

o How WMSD was defined across studies

o Whether data extraction was done independently by two reviewers

o Adjusted vs. crude effect sizes

Response: Thank you for your valuable concerns regarding the data extraction variables. However, we have previously specified in the inclusion criteria that WMSD is defined as studies reporting the prevalence of WMSD in at least one body area (neck, shoulder, elbow, wrist/hand, upper back, lower back, hip/thigh, knee, and foot/ankle) within the past year. Additionally, we have provided an operational definition. In the "Outcome Measurement" subsection, we clarified that a pooled adjusted odds ratio (AOR) was calculated to assess the relationship between WMSDs and their associated factors. Furthermore, we indicated that data extraction was conducted independently by two researchers (ADD & AB), and any disagreements were resolved by a third author (EB & TT), as detailed in the "Data Extraction, Study Quality, and Risk of Bias Assessment" subsection.

e. No Handling of Variation Between Studies

• WMSD definitions, measurement tools, and anatomical regions may differ widely.

• No subgroup analyses (e.g., by region, measurement tool, year of study), which could help explain heterogeneity.

Response: Thank you for highlighting the handling of variation between studies. We addressed the variation in WMSD definitions, and anatomical regions in our predefined inclusion criteria, which specify studies reporting the prevalence of WMSD in at least one body area (neck, shoulder, elbow, wrist/hand, upper back, lower back, hip/thigh, knee, and foot/ankle) within the past year, as well as in the operational definition. We conducted subgroup analyses based on the number of body areas affected by WMSDs. Additionally, we previously performed subgroup analyses by region and publication year. In response to your constructive recommendation, we have now included subgroup analyses by measurement tool as well. We appreciate your input, which has contributed to the robustness of our analysis.

4. Comment: Results

Strengths

• Provides pooled sample size and number of included studies.

• Reports pooled prevalence with confidence intervals.

• Identifies significant associated factors with pooled odds ratios.

Limitations

a. High Heterogeneity Likely

• Although I² is mentioned, its value is not reported.

• Prevalence studies typically show high heterogeneity; without subgroup/exploratory analyses, results may be unreliable.

Response: Thank you for your concern regarding heterogeneity. The I² value is indeed mentioned in the previously submitted manuscript under the "Meta-Analysis" subsection, where it is reported as 18.8%. We also conducted subgroup analyses based on region, sample size, publication year, and the number of anatomical areas. In light of your insightful feedback, we have additionally performed subgroup analyses by measurement tool. We appreciate your input, which has enhanced the rigor of our analysis.

b. No Visuals Reported

• Forest plot, funnel plot, trim-and-fill output, and PRISMA flow diagram are essential but not mentioned or described.

c. Limited Examination of Associated Factors

• Only four factors identified; unclear if others were tested (e.g., BMI, working hours, shift work, patient-handling practices).

d. No Sensitivity Analysis Results Reported

• Stated as conducted but results not summarized.

Response: Thank you for your feedback. The forest plot, funnel plot, trim-and-fill analysis, and PRISMA flow diagram were indeed described in the first section of the results and the publication bias assessment. Additionally, sensitivity analysis result was summarized or reported clearly under sensitivity analysis sub section. Moreover, A total of 14 variables were examined in this study, including age, sex, smoking status, working in awkward positions, bending or twisting during work, assistance from a colleague, job stress, job satisfaction, sleep disturbance, prolonged static postures, work experience, weekly working hours, and night shifts. These variables are detailed in the accompanying Table 2. Out of these, only four—sex, job stress, prolonged standing, and work experience—were found to have a significant association with work-related musculoskeletal disorders (WMSDs). Thank you again for bringing these points to our attention. Your insights are invaluable for improving the clarity and comprehensiveness of the study.

5. Comment: Discussion

Strengths

• Conclusion aligns with findings.

• Suggests practical implications (e.g., focus on reducing stress and standing time).

Limitations

• Does not compare pooled estimates with global or African data.

• Does not address:

o Potential reverse causation (cross-sectional designs dominate this field).

o Measurement bias (self-reported outcomes).

o Differences in workplace ergonomics across studies.

Response: Thank you for your valuable feedback on the discussion section. We appreciate your insights regarding its strengths and limitations. We acknowledge the points raised about the comparison with global and African data, potential reverse causation, measurement bias related to self-reported outcomes, and the variations in workplace ergonomics across studies. These aspects have been addressed in the revised manuscript

6. Comment: Conclusion

Strengths

• Acknowledges high prevalence and identifies actionable factors.

Limitations

• Recommendations are generic and lack specificity (e.g., ergonomic programs, workload redesign, staffing ratio improvement).

• Does not address research implications, such as the need for longitudinal or intervention studies.

Response: Thank you for your thoughtful comments regarding the conclusion of the study. We appreciate your recognition of the strengths. In response to your feedback on the limitations, we have revised the recommendations to include more specific strategies, such as the implementation of ergonomic training, improvements in staffing ratios, proper body mechanics etc. Additionally, we have incorporated a discussion on the research implications, emphasizing the need for longitudinal or intervention studies to further explore these issues. Your insights have greatly contributed to enhancing the clarity and depth of our conclusions. Thank you once again for your valuable feedback.

7. Comment: Overall Strengths of the Study

• Addresses an important occupational health issue.

• Uses meta-analysis to synthesize evidence valuable for policymaking.

• Includes multiple databases and unpublished studies.

• Demonstrates methodological awareness (heterogeneity, publication bias).

Overall Limitations and Areas for Improvement

Methodological Limitations

• Insufficient reporting on search strategy and risk-of-bias assessment.

• Unclear adherence to PRISMA guidelines.

• No subgroup or meta-regression analyses.

• Unclear handling of inconsistent definitions of WMSD.

• High heterogeneity likely undermines pooled prevalence reliability.

Analytical Limitations

• Effect estimates may be confounded due to lack of adjusted ORs.

• No weighting or discussion of study quality.

Interpretation Limitations

• Overgeneralizes findings without accounting for workplace variability across Ethiopia.

Response: Thank you for your thorough evaluation of the study's strengths and limitations. We truly appreciate your insights on the methodological and analytical aspects, as well as the interpretation limitations. We have taken your feedback to heart and have addressed the issues raised, including enhancing the reporting on the search strategy and risk-of-bias assessment, clarifying adherence to PRISMA guidelines, and discussing the handling of inconsistent definitions of WMSD in the revised manuscript. Thank you again for your constructive comments

8. Comment: Summary

The study provides useful pooled estimates of WMSDs among nurses in Ethiopia, showing a high prevalence (61.47%) and identifying significant associated factors. However, methodological weaknesses especially incomplete reporting of search strategy, lack of study quality assessment, unclear heterogeneity handling, and limited discussion of contextual factors reduce confidence in the pooled estimates. Strengthening transparency, performing subgroup analyses, and adhering strictly to PRISMA standards would significantly improve the study’s rigor and credibility.

Response: Thank you for your thoughtful summary and feedback on the study. We appreciate your recognition of the useful pooled estimates and associated factors identified. We acknowledge the methodological weaknesses mentioned, specifically regarding the reporting of the search strategy, study quality assessment, handling of heterogeneity, and discussion of contextual factors. We have made revisions to address these issues, aiming to strengthen transparency, perform subgroup analyses, and adhere more closely to PRISMA standards in the revise

Attachment Submitted filename: Response to Reviewers.docx

# Peer review: Decision Letter 1

**DOI:** 10.1371/journal.pone.0354484.r003

**Authors:** Sohel Ahmed

25 Mar 2026

PONE-D-25-47479R1

A systematic review and meta-analysis on the epidemiology of work-related musculoskeletal disorders among nurses in Ethiopia

PLOS One

Dear Dr.  Demeke,

Thank you for submitting your manuscript to PLOS ONE. After careful consideration, we feel that it has merit but does not fully meet PLOS ONE’s publication criteria as it currently stands. Therefore, we invite you to submit a revised version of the manuscript that addresses the points raised during the review process.

Please submit your revised manuscript by May 09 2026 11:59PM. If you will need more time than this to complete your revisions, please reply to this message or contact the journal office at plosone@plos.org. When you're ready to submit your revision, log on to https://www.editorialmanager.com/pone/ and select the 'Submissions Needing Revision' folder to locate your manuscript file.

Please include the following items when submitting your revised manuscript:

- A letter that responds to each point raised by the academic editor and reviewer(s). You should upload this letter as a separate file labeled 'Response to Reviewers'.
- A marked-up copy of your manuscript that highlights changes made to the original version. You should upload this as a separate file labeled 'Revised Manuscript with Track Changes'.
- An unmarked version of your revised paper without tracked changes. You should upload this as a separate file labeled 'Manuscript'.

If you would like to make changes to your financial disclosure, please include your updated statement in your cover letter. Guidelines for resubmitting your figure files are available below the reviewer comments at the end of this letter.

If applicable, we recommend that you deposit your laboratory protocols in protocols.io to enhance the reproducibility of your results. Protocols.io assigns your protocol its own identifier (DOI) so that it can be cited independently in the future. For instructions see: https://journals.plos.org/plosone/s/submission-guidelines#loc-laboratory-protocols. Additionally, PLOS ONE offers an option for publishing peer-reviewed Lab Protocol articles, which describe protocols hosted on protocols.io. Read more information on sharing protocols at https://plos.org/protocols?utm_medium=editorial-email&utm_source=authorletters&utm_campaign=protocols.

As the corresponding author, your ORCID iD is verified in the submission system and will appear in the published article. PLOS supports the use of ORCID, and we encourage all coauthors to register for an ORCID iD and use it as well. Please encourage your coauthors to verify their ORCID iD within the submission system before final acceptance, as unverified ORCID iDs will not appear in the published article. *Only*  the individual author can complete the verification step; PLOS staff *cannot*  verify ORCID iDs on behalf of authors.

We look forward to receiving your revised manuscript.

Kind regards,

Sohel Ahmed, BPT, MPT, MDMR

Academic Editor

PLOS One

Journal Requirements:

1. If the reviewer comments include a recommendation to cite specific previously published works, please review and evaluate these publications to determine whether they are relevant and should be cited. There is no requirement to cite these works unless the editor has indicated otherwise.

2. Please review your reference list to ensure that it is complete and correct. If you have cited papers that have been retracted, please include the rationale for doing so in the manuscript text, or remove these references and replace them with relevant current references. Any changes to the reference list should be mentioned in the rebuttal letter that accompanies your revised manuscript. If you need to cite a retracted article, indicate the article’s retracted status in the References list and also include a citation and full reference for the retraction notice.

[Note: HTML markup is below. Please do not edit.]

Reviewer's Responses to Questions

**Comments to the Author**

1. If the authors have adequately addressed your comments raised in a previous round of review and you feel that this manuscript is now acceptable for publication, you may indicate that here to bypass the “Comments to the Author” section, enter your conflict of interest statement in the “Confidential to Editor” section, and submit your "Accept" recommendation.

Reviewer #2: All comments have been addressed

Reviewer #3: All comments have been addressed

**********

2. Is the manuscript technically sound, and do the data support the conclusions?

The manuscript must describe a technically sound piece of scientific research with data that supports the conclusions. Experiments must have been conducted rigorously, with appropriate controls, replication, and sample sizes. The conclusions must be drawn appropriately based on the data presented.

Reviewer #2: Yes

Reviewer #3: Yes

**********

3. Has the statistical analysis been performed appropriately and rigorously?

Reviewer #2: Yes

Reviewer #3: Yes

**********

4. Have the authors made all data underlying the findings in their manuscript fully available?

The PLOS Data policy requires authors to make all data underlying the findings described in their manuscript fully available without restriction, with rare exception (please refer to the Data Availability Statement in the manuscript PDF file). The data should be provided as part of the manuscript or its supporting information, or deposited to a public repository. For example, in addition to summary statistics, the data points behind means, medians and variance measures should be available. If there are restrictions on publicly sharing data—e.g. participant privacy or use of data from a third party—those must be specified.

Reviewer #2: Yes

Reviewer #3: Yes

**********

5. Is the manuscript presented in an intelligible fashion and written in standard English?

PLOS ONE does not copyedit accepted manuscripts, so the language in submitted articles must be clear, correct, and unambiguous. Any typographical or grammatical errors should be corrected at revision, so please note any specific errors here.

Reviewer #2: Yes

Reviewer #3: Yes

**********

6. Review Comments to the Author

Please use the space provided to explain your answers to the questions above. You may also include additional comments for the author, including concerns about dual publication, research ethics, or publication ethics. (Please upload your review as an attachment if it exceeds 20,000 characters)

Reviewer #2: The authors have made substantial and sincere efforts to address the reviewers’ comments. The revised manuscript demonstrates marked improvement in transparency, methodological reporting, and discussion depth. Many of the major concerns raised in the initial review particularly those related to PRISMA adherence, search strategy reporting, subgroup analyses, and contextual discussion—have now been addressed.

However, several important issues remain, mainly related to interpretation of heterogeneity, pooling of adjusted odds ratios, and internal consistency. These issues do not invalidate the study but should be addressed before final acceptance.

Major Comments

1. PRISMA Compliance and Reporting Transparency — Largely Resolved

✔ The authors now explicitly state adherence to PRISMA 2020, provide a flow diagram, and submit a PRISMA checklist.

✔ Search dates, databases, and inclusion criteria are clearer.

✔ Supplementary materials now include detailed search strategies.

Remaining issue:

• Although PRISMA compliance is stated, the manuscript would benefit from a brief explicit statement confirming that all PRISMA 2020 items were met, with reference to the submitted checklist.

Recommendation: Minor clarification only.

2. Search Strategy and Reproducibility — Mostly Resolved

✔ Full electronic search strategies are provided as supplementary material.

✔ Language and date restrictions are now explicitly stated.

✔ Justification for Google Scholar use is included.

Remaining issue:

• The authors explicitly state that reference list screening and manual searching were not performed. While acceptable, this should be acknowledged as a limitation, as it may affect completeness.

Recommendation: Add one sentence in the limitations section.

3. Study Quality Assessment and Risk of Bias — Partially Resolved

✔ Use of the JBI critical appraisal checklist is appropriate and clearly described.

✔ Independent assessment by multiple reviewers is reported.

Concern remains:

• All included studies are classified as “low risk of bias,” which is unusual for cross-sectional observational studies.

• The justification provided is brief and may not fully convince readers.

Recommendation:

• Temper the language to acknowledge that “low risk” reflects methodological adequacy within JBI criteria, but residual bias typical of cross-sectional designs cannot be excluded.

4. Heterogeneity and Subgroup Analyses — Improved but Interpretation Needs Caution

✔ Subgroup analyses by region, year, sample size, body areas, and measurement tool have been added and strengthen the analysis.

✔ Sensitivity analyses are reported.

Critical issue:

• Some pooled associations (e.g., prolonged standing, sleep disturbance) show very high heterogeneity (I² > 85–90%), yet are still presented as significant findings.

Recommendation:

• Interpret these pooled estimates more cautiously and explicitly state that high heterogeneity limits confidence in these specific associations.

• Avoid emphasizing these findings in the conclusion.

5. Pooling of Adjusted Odds Ratios — Still a Methodological Weakness

The manuscript pools adjusted odds ratios from studies with different covariate sets, which may compromise internal validity.

Although the authors now acknowledge this issue, the limitation remains methodologically important.

Recommendation:

• Explicitly state that pooled AORs should be interpreted as approximate summaries, not precise effect estimates.

• Consider adding a brief justification for why meta-regression was not feasible (e.g., limited number of studies per factor).

6. Language Suggesting Causality — Mostly Resolved

✔ The authors have revised much of the language to reflect associations rather than causation.

✔ The observational nature of the evidence is now acknowledged.

Minor remaining issue:

• A few phrases in the Discussion and Conclusion still imply risk reduction or prevention effects.

Recommendation: Final language polishing to ensure consistent non-causal framing.

Minor Comments

1. Internal consistency:

o Double-check numerical consistency between the PRISMA flow diagram and the Results text (earlier inconsistencies were corrected, but a final check is advised).

2. Terminology:

o Consistent use of WMSDs is improved; continue to ensure uniform usage across figures and tables.

3. Tables:

o Table 2 would benefit from a footnote reminding readers that pooled AORs are derived from heterogeneous adjustment models.

Reviewer #3: Regarding the introduction, strong epidemiological justification, clear research gap, comprehensive risk factor coverage, and strong contextual relevance. If possible, reduce the Redundancy.

Protocol registration, PRISMA adherence, rigorous statistical methods, dual-review processes are major strengths. Kindly, describe the methods for pooling adjusted odds ratios (e.g., log transformation, heterogeneity handling, model selection).

**********

7. PLOS authors have the option to publish the peer review history of their article (what does this mean?). If published, this will include your full peer review and any attached files.

If you choose “no”, your identity will remain anonymous but your review may still be made public.

**Do you want your identity to be public for this peer review?**  For information about this choice, including consent withdrawal, please see our Privacy Policy.

Reviewer #2: No

Reviewer #3: **Yes:**  Dr. Lareen Magdi El-Sayed Abo-Seif

**********

[NOTE: If reviewer comments were submitted as an attachment file, they will be attached to this email and accessible via the submission site. Please log into your account, locate the manuscript record, and check for the action link "View Attachments". If this link does not appear, there are no attachment files.]

To ensure your figures meet our technical requirements, please review our figure guidelines: https://journals.plos.org/plosone/s/figures

You may also use PLOS’s free figure tool, NAAS, to help you prepare publication quality figures: https://journals.plos.org/plosone/s/figures#loc-tools-for-figure-preparation.

NAAS will assess whether your figures meet our technical requirements by comparing each figure against our figure specifications.

Attachment Submitted filename: New Microsoft Word Document (2).docx

# Author comment: Author response to Decision Letter 2

**DOI:** 10.1371/journal.pone.0354484.r004

27 Mar 2026

Editor’s comment

1. Comment: If the reviewer comments include a recommendation to cite specific previously published works, please review and evaluate these publications to determine whether they are relevant and should be cited. There is no requirement to cite these works unless the editor has indicated otherwise.

Response: Thank you for your guidance. We appreciate your emphasis on the importance of relevant citations. However, we would like to clarify that the reviewer did not recommend any specific previously published works for us to cite

2. Comment: Please review your reference list to ensure that it is complete and correct. If you have cited papers that have been retracted, please include the rationale for doing so in the manuscript text, or remove these references and replace them with relevant current references. Any changes to the reference list should be mentioned in the rebuttal letter that accompanies your revised manuscript. If you need to cite a retracted article, indicate the article’s retracted status in the References list and also include a citation and full reference for the retraction notice.

Response: Thank you for this request. We reviewed the reference list and confirm that it is complete and correct. In addition, no retracted papers are cited in our manuscript; therefore no changes were required.

Review Comments to the Author

Reviewer #2

Overall Evaluation

1. Comment: The authors have made substantial and sincere efforts to address the reviewers’ comments. The revised manuscript demonstrates marked improvement in transparency, methodological reporting, and discussion depth. Many of the major concerns raised in the initial review particularly those related to PRISMA adherence, search strategy reporting, subgroup analyses, and contextual discussion—have now been addressed.

However, several important issues remain, mainly related to interpretation of heterogeneity, pooling of adjusted odds ratios, and internal consistency. These issues do not invalidate the study but should be addressed before final acceptance.

Response: Thank you for your careful review and encouraging comments. We appreciate that you recognize the improvements in transparency, methods reporting, and discussion. We have carefully considered the remaining issues you raised—interpretation of heterogeneity, pooling of adjusted odds ratios, and internal consistency—and have addressed them in the revised manuscript.

Major comments

2. Comment: PRISMA Compliance and Reporting Transparency — Largely Resolved

✔ The authors now explicitly state adherence to PRISMA 2020, provide a flow diagram, and submit a PRISMA checklist.

✔ Search dates, databases, and inclusion criteria are clearer.

✔ Supplementary materials now include detailed search strategies.

Remaining issue:

• Although PRISMA compliance is stated, the manuscript would benefit from a brief explicit statement confirming that all PRISMA 2020 items were met, with reference to the submitted checklist.

Recommendation: Minor clarification only

Response: Thank you for your confirmation and constructive feedback.

We have added a brief, explicit statement to the revised manuscript clarifying PRISMA 2020 compliance.

3. Comment: Search Strategy and Reproducibility — Mostly Resolved

✔ Full electronic search strategies are provided as supplementary material.

✔ Language and date restrictions are now explicitly stated.

✔ Justification for Google Scholar use is included.

Remaining issue:

• The authors explicitly state that reference list screening and manual searching were not performed. While acceptable, this should be acknowledged as a limitation, as it may affect completeness. Recommendation: Add one sentence in the limitations section.

Response: Thank you for your confirmation and constructive feedback. We have addressed the remaining comment by adding the following sentence to the Limitations section: "we did not perform reference-list screening or manual searches of identified articles; this may have reduced the completeness of study identification" Thank you once again for your helpful suggestions

4. Comment: Study Quality Assessment and Risk of Bias — Partially Resolved

✔ Use of the JBI critical appraisal checklist is appropriate and clearly described.

✔ Independent assessment by multiple reviewers is reported.

Concern remains:

• All included studies are classified as “low risk of bias,” which is unusual for cross-sectional observational studies.

• The justification provided is brief and may not fully convince readers.

Recommendation:

• Temper the language to acknowledge that “low risk” reflects methodological adequacy within JBI criteria, but residual bias typical of cross-sectional designs cannot be excluded.

Response: Thank you very much for this helpful observation. We have addressed the concern in the revised manuscript under “Quality assessment and risk of bias” section.

5. Comment: Heterogeneity and Subgroup Analyses — Improved but Interpretation Needs Caution

✔ Subgroup analyses by region, year, sample size, body areas, and measurement tool have been added and strengthen the analysis.

✔ Sensitivity analyses are reported.

Critical issue:

• Some pooled associations (e.g., prolonged standing, sleep disturbance) show very high heterogeneity (I² > 85–90%), yet are still presented as significant findings.

Recommendation:

• Interpret these pooled estimates more cautiously and explicitly state that high heterogeneity limits confidence in these specific associations.

• Avoid emphasizing these findings in the conclusion

Response: Thank you for this important guidance. In the previously submitted manuscript we explicitly stated that high heterogeneity limits confidence in these specific associations (lines 340–343, 352–354 etc). We have tempered the interpretation of these findings throughout the Discussion to reflect this uncertainty. However, we note that outright removal of these results from the Conclusion is not scientifically warranted, because the pooled estimates—despite high heterogeneity—still provide useful evidence that we report transparently alongside caveats about their limited confidence. We therefore retained the findings in the Conclusion. Thank you once again for your helpful suggestions.

6. Comment: Pooling of Adjusted Odds Ratios — Still a Methodological Weakness

The manuscript pools adjusted odds ratios from studies with different covariate sets, which may compromise internal validity.

Although the authors now acknowledge this issue, the limitation remains methodologically important.

Recommendation:

• Explicitly state that pooled AORs should be interpreted as approximate summaries, not precise effect estimates.

• Consider adding a brief justification for why meta-regression was not feasible (e.g., limited number of studies per factor)

Response: Thank you for this important methodological point. We have clarified in the revised manuscript under statistical analysis section that pooled adjusted odds ratios (PAORs) are presented as approximate summary measures—not precise effect estimates—because included studies adjusted for different covariate sets. We also explain in the Strengths and Limitations section that meta‑regression was not feasible due to the limited number of studies reporting comparable adjusted estimates per exposure

7. Comment: Language Suggesting Causality — Mostly Resolved

✔ The authors have revised much of the language to reflect associations rather than causation.

✔ The observational nature of the evidence is now acknowledged.

Minor remaining issue:

• A few phrases in the Discussion and Conclusion still imply risk reduction or prevention effects.

Recommendation: Final language polishing to ensure consistent non-causal framing.

Response: Thank you for this helpful suggestion. We have completed a final language polish to remove causal wording and ensure consistent non‑causal framing throughout the Discussion and Conclusion.

Minor comments

8. Comment: Internal consistency:

o Double-check numerical consistency between the PRISMA flow diagram and the Results text (earlier inconsistencies were corrected, but a final check is advised)

Response: Thank you for this careful check. We have performed a final verification and confirm that numerical counts in the PRISMA flow diagram and the Results text are now consistent

9. Comment: Terminology:

o Consistent use of WMSDs is improved; continue to ensure uniform usage across figures and tables

Response: Thank you. We have standardized the terminology throughout the manuscript and confirm that 'WMSDs' is used uniformly across the text, figures, and tables.

10. Comment: Tables:

o Table 2 would benefit from a footnote reminding readers that pooled AORs are derived from heterogeneous adjustment models

Response: Thank you. We have added a statement below Table 2 noting that PAOR should be interpreted as approximate summaries, rather than precise effect estimates due to substantial variability of covariates observed across studies.

Reviewer #3

1. Comment: Regarding the introduction, strong epidemiological justification, clear research gap, comprehensive risk factor coverage, and strong contextual relevance. If possible, reduce the Redundancy.

Protocol registration, PRISMA adherence, rigorous statistical methods, dual-review processes are major strengths. Kindly, describe the methods for pooling adjusted odds ratios (e.g., log transformation, heterogeneity handling, model selection).

Response: Thank you very much for these positive and constructive comments. We appreciate your recognition of the manuscript’s epidemiological justification, clear research gap, comprehensive risk‑factor coverage, and contextual relevance, as well as the strengths you noted (protocol registration, PRISMA adherence, rigorous statistical methods, and dual independent review).

Regarding the request to describe methods for pooling adjusted odds ratios, we briefly described this in the Methods under the Statistical analysis subsection. Thank you once again — we believe your input has substantially improved the manuscript.

Attachment Submitted filename: Response_to_Reviewers_auresp_2.docx

# Peer review: Decision Letter 2

**DOI:** 10.1371/journal.pone.0354484.r005

**Authors:** Sohel Ahmed

18 Apr 2026

PONE-D-25-47479R2

A systematic review and meta-analysis on the epidemiology of work-related musculoskeletal disorders among nurses in Ethiopia

PLOS One

Dear Dr.  Demeke,

Thank you for submitting your manuscript to PLOS ONE. After careful consideration, we feel that it has merit but does not fully meet PLOS ONE’s publication criteria as it currently stands. Therefore, we invite you to submit a revised version of the manuscript that addresses the points raised during the review process.

Please submit your revised manuscript by Jun 02 2026 11:59PM. If you will need more time than this to complete your revisions, please reply to this message or contact the journal office at plosone@plos.org. When you're ready to submit your revision, log on to https://www.editorialmanager.com/pone/ and select the 'Submissions Needing Revision' folder to locate your manuscript file.

Please include the following items when submitting your revised manuscript:

- A letter that responds to each point raised by the academic editor and reviewer(s). You should upload this letter as a separate file labeled 'Response to Reviewers'.
- A marked-up copy of your manuscript that highlights changes made to the original version. You should upload this as a separate file labeled 'Revised Manuscript with Track Changes'.
- An unmarked version of your revised paper without tracked changes. You should upload this as a separate file labeled 'Manuscript'.

If you would like to make changes to your financial disclosure, please include your updated statement in your cover letter. Guidelines for resubmitting your figure files are available below the reviewer comments at the end of this letter.

If applicable, we recommend that you deposit your laboratory protocols in protocols.io to enhance the reproducibility of your results. Protocols.io assigns your protocol its own identifier (DOI) so that it can be cited independently in the future. For instructions see: https://journals.plos.org/plosone/s/submission-guidelines#loc-laboratory-protocols. Additionally, PLOS ONE offers an option for publishing peer-reviewed Lab Protocol articles, which describe protocols hosted on protocols.io. Read more information on sharing protocols at https://plos.org/protocols?utm_medium=editorial-email&utm_source=authorletters&utm_campaign=protocols.

As the corresponding author, your ORCID iD is verified in the submission system and will appear in the published article. PLOS supports the use of ORCID, and we encourage all coauthors to register for an ORCID iD and use it as well. Please encourage your coauthors to verify their ORCID iD within the submission system before final acceptance, as unverified ORCID iDs will not appear in the published article. *Only*  the individual author can complete the verification step; PLOS staff *cannot*  verify ORCID iDs on behalf of authors.

We look forward to receiving your revised manuscript.

Kind regards,

Sohel Ahmed, BPT, MPT, MDMR

Academic Editor

PLOS One

Journal Requirements:

1. If the reviewer comments include a recommendation to cite specific previously published works, please review and evaluate these publications to determine whether they are relevant and should be cited. There is no requirement to cite these works unless the editor has indicated otherwise.

2. Please review your reference list to ensure that it is complete and correct. If you have cited papers that have been retracted, please include the rationale for doing so in the manuscript text, or remove these references and replace them with relevant current references. Any changes to the reference list should be mentioned in the rebuttal letter that accompanies your revised manuscript. If you need to cite a retracted article, indicate the article’s retracted status in the References list and also include a citation and full reference for the retraction notice.

[Note: HTML markup is below. Please do not edit.]

Reviewer's Responses to Questions

**Comments to the Author**

1. If the authors have adequately addressed your comments raised in a previous round of review and you feel that this manuscript is now acceptable for publication, you may indicate that here to bypass the “Comments to the Author” section, enter your conflict of interest statement in the “Confidential to Editor” section, and submit your "Accept" recommendation.

Reviewer #2: All comments have been addressed

**********

2. Is the manuscript technically sound, and do the data support the conclusions?

The manuscript must describe a technically sound piece of scientific research with data that supports the conclusions. Experiments must have been conducted rigorously, with appropriate controls, replication, and sample sizes. The conclusions must be drawn appropriately based on the data presented.

Reviewer #2: Yes

**********

3. Has the statistical analysis been performed appropriately and rigorously?

Reviewer #2: Yes

**********

4. Have the authors made all data underlying the findings in their manuscript fully available?

The PLOS Data policy requires authors to make all data underlying the findings described in their manuscript fully available without restriction, with rare exception (please refer to the Data Availability Statement in the manuscript PDF file). The data should be provided as part of the manuscript or its supporting information, or deposited to a public repository. For example, in addition to summary statistics, the data points behind means, medians and variance measures should be available. If there are restrictions on publicly sharing data—e.g. participant privacy or use of data from a third party—those must be specified.

Reviewer #2: Yes

**********

5. Is the manuscript presented in an intelligible fashion and written in standard English?

PLOS ONE does not copyedit accepted manuscripts, so the language in submitted articles must be clear, correct, and unambiguous. Any typographical or grammatical errors should be corrected at revision, so please note any specific errors here.

Reviewer #2: Yes

**********

6. Review Comments to the Author

Please use the space provided to explain your answers to the questions above. You may also include additional comments for the author, including concerns about dual publication, research ethics, or publication ethics. (Please upload your review as an attachment if it exceeds 20,000 characters)

Reviewer #2: Overall assessment

The paper addresses an important occupational health issue and has several strengths: protocol registration, PRISMA-based reporting, multiple database searching, duplicate screening/quality appraisal, subgroup analysis, publication-bias assessment, and sensitivity analysis. The study also provides a useful pooled estimate for WMSDs among nurses in Ethiopia.

That said, the manuscript still has problems in four main areas:

• English language and grammar

• Overstatement of findings in some places

• Methodological inconsistency in interpretation of pooled adjusted odds ratios

• Need for tighter scientific writing, especially in Abstract, Discussion, and Conclusion

Major issues

1. Language needs substantial polishing

The manuscript still contains many grammatical and style problems that reduce readability and credibility.

Examples:

“no comprehensive meta-analysis have been done” should be “has been done.”

“Joana Brigg Institute” should be “Joanna Briggs Institute.”

“≥ 5 year of work experience of” is grammatically incorrect.

“The protocol … has been registered … codeCRD420251136383.We didn’t make an amendment…” is awkward and not journal-ready.

“two researchers independently evaluate” should be past tense.

This is not just cosmetic. The paper needs a full professional English edit.

2. The conclusion still overreaches

The paper repeatedly acknowledges that pooled AORs are only approximate summaries because studies adjusted for different covariates, and that some associations had high heterogeneity.

However, the conclusion still moves too far toward intervention language:

“Reducing job stress and prolonged standing…” in the abstract conclusion

“it may be important to improve staffing ratios…” in the final conclusion

For a review based entirely on observational studies, the wording should stay with:

“were associated with”

“may warrant attention”

“should be interpreted cautiously”

“may inform occupational health planning”

Not with implied preventive effect unless clearly framed as a recommendation rather than evidence of effect.

3. High-heterogeneity associations are still emphasized too strongly

The manuscript appropriately notes high heterogeneity for job stress and especially prolonged standing.

But these factors are still emphasized in the abstract and conclusion almost as if they were robust effect estimates. That is too strong, especially for:

Prolonged standing: I² = 91.4%

Job stress: I² = 70.8%

These should remain in the results, but in the abstract and conclusion the authors should make clear that these associations were observed with substantial between-study variability.

4. The Discussion needs more scientific restraint Some explanatory statements are too assertive or simplistic.

For example, the sex explanation relies heavily on biological reasoning and muscle mass/testosterone differences.

That is possible, but it reads as overly deterministic. The discussion should also acknowledge:

• role differences and task allocation

• domestic workload

• ergonomics

• reporting differences

• workplace context

A more balanced interpretation would be stronger scientifically.

5. Some claims in the Introduction are overstated or awkwardly phrased

Examples:

• “This variability and scarcity of clear epidemiological understanding deterred the design of diagnostic criteria, the establishment of specialized services, and recruitment in intervention trials.”

This feels exaggerated and probably unsupported.

• “This study is a reliable, evidence-based basis…” should be rewritten more modestly.

The introduction should be tighter and more evidence-led.

Methodological concerns

6) Pooling adjusted odds ratios remains a limitation The authors now acknowledge that PAORs are approximate because covariates differed across studies. That is good.

Still, the manuscript should be even clearer that:

• pooled adjusted effects across heterogeneous models are not equivalent to pooled effects from harmonized adjustment sets

• this reduces comparability and internal validity

• these results are exploratory summaries, not precise quantitative causal estimates

This caution should appear not only in Methods and Table 2 footnote, but also briefly in the Abstract or Conclusion.

7) Some factor interpretations are statistically awkward For work experience:

• PAOR = 1.58 (1.08–2.31), I² = 38.6%, P = 0.196

This appears to mix the heterogeneity p-value with inferential interpretation. The result is statistically significant based on the confidence interval excluding 1, but the narrative should avoid confusing readers by presenting the heterogeneity p-value next to the effect without clarification.

Also, in Table 2 and surrounding text, formatting and presentation should be standardized to avoid ambiguity.

Internal consistency and reporting

8) Scope of outcome needs clearer wording

The paper is framed as WMSDs among nurses, but several included studies appear focused on single body regions, especially low back pain or ankle-foot pain.

The authors partly address this by subgrouping according to number of body areas assessed.

Still, they should state more explicitly that the pooled estimate combines studies with different anatomical scopes, which may affect comparability.

9. Reference and citation quality should be rechecked A few citations look weak or mismatched:

Reference 30 is a study among bank workers in Ethiopia, yet it is cited for work-related musculoskeletal disorder definition/associated factors in places where a more direct occupational health or nursing source would be stronger.

Some supporting statements in the discussion would benefit from more directly relevant sources and less generic explanation.

Minor issues

10)) Title and abstract wording

The title is acceptable, but the abstract needs line editing for grammar and flow.

For example:

• “Result” should be “Results”

• “its associated factors” should be “associated factors”

• avoid recommendation-style language in the conclusion

11. Tables need cleanup

Table 1 and Table 2 are useful, but formatting is rough and should be standardized for journal submission.

Examples:

• inconsistent spacing

• awkward variable labels

• inconsistent capitalization

• some row labels are not reader-friendly

12. Strengths and limitations

section should be rewritten The section is useful, but the language is weak and repetitive.

For instance:

• “intensifies transparency” is not natural scientific English

• “negate WMSDs” in the conclusion is incorrect usage

**********

7. PLOS authors have the option to publish the peer review history of their article (what does this mean?). If published, this will include your full peer review and any attached files.

If you choose “no”, your identity will remain anonymous but your review may still be made public.

**Do you want your identity to be public for this peer review?**  For information about this choice, including consent withdrawal, please see our Privacy Policy.

Reviewer #2: **Yes:**  Dr. Ali Daifallah abousoliman

**********

[NOTE: If reviewer comments were submitted as an attachment file, they will be attached to this email and accessible via the submission site. Please log into your account, locate the manuscript record, and check for the action link "View Attachments". If this link does not appear, there are no attachment files.]

To ensure your figures meet our technical requirements, please review our figure guidelines: https://journals.plos.org/plosone/s/figures

You may also use PLOS’s free figure tool, NAAS, to help you prepare publication quality figures: https://journals.plos.org/plosone/s/figures#loc-tools-for-figure-preparation.

NAAS will assess whether your figures meet our technical requirements by comparing each figure against our figure specifications.

Attachment Submitted filename: New Microsoft Word Document (5).docx

# Author comment: Author response to Decision Letter 3

**DOI:** 10.1371/journal.pone.0354484.r006

31 May 2026

Editor’s Comment

1. Comment: If the reviewer comments include a recommendation to cite specific previously published works, please review and evaluate these publications to determine whether they are relevant and should be cited. There is no requirement to cite these works unless the editor has indicated otherwise.

Response: Thank you for your guidance. We appreciate your emphasis on the importance of relevant citations. However, we would like to clarify that the reviewer did not recommend any specific previously published works for us to cite

2. Comment: Please review your reference list to ensure that it is complete and correct. If you have cited papers that have been retracted, please include the rationale for doing so in the manuscript text, or remove these references and replace them with relevant current references. Any changes to the reference list should be mentioned in the rebuttal letter that accompanies your revised manuscript. If you need to cite a retracted article, indicate the article’s retracted status in the References list and also include a citation and full reference for the retraction notice.

Response: Thank you for this request. We reviewed the reference list and confirmed that it is complete and correct. In addition, no retracted papers are cited in our manuscript; therefore, no changes were required.

Review Comments

Overall assessment

1. Comment: The paper addresses an important occupational health issue and has several strengths: protocol registration, PRISMA-based reporting, multiple database searching, duplicate screening/quality appraisal, subgroup analysis, publication-bias assessment, and sensitivity analysis. The study also provides a useful pooled estimate for WMSDs among nurses in Ethiopia. That said, the manuscript still has problems in four main areas: • English language and grammar

• Overstatement of findings in some places

• Methodological inconsistency in interpretation of pooled adjusted odds ratios

• Need for tighter scientific writing, especially in Abstract, Discussion, and Conclusion

Response: Thank you for the helpful feedback and for recognizing the strengths of our manuscript. We agree that the issues you identified—English language and grammar, overstatement of findings, inconsistencies in the interpretation of the pooled adjusted odds ratios, and the need for tighter scientific writing—require improvement. We have addressed each of these concerns in the revised manuscript, including improving the overall writing quality (particularly in the Abstract, Discussion, and Conclusion), carefully revising the interpretation and reporting of pooled adjusted odds ratios to ensure methodological consistency, and revising statements where the strength of conclusions may have been overstated.

# Major issues

2. Comment: Language needs substantial polishing

The manuscript still contains many grammatical and style problems that reduce readability and credibility.

Examples:

“No comprehensive meta-analysis has been done” should be “has been done.” “Joana Brigg Institute” should be “Joanna Briggs Institute.”

“≥ 5 years of work experience" is grammatically incorrect.

“The protocol … has been registered … codeCRD420251136383. We didn’t make an amendment…” is awkward and not journal-ready.

“Two researchers independently evaluate” should be past tense.

This is not just cosmetic. The paper needs a full professional English edit.

Response: Thank you for your comment and for the specific examples. We agree that the manuscript requires substantial language polishing to improve readability and enhance its credibility. In the revised manuscript, we have corrected the grammatical and stylistic issues you highlighted (e.g., subject–verb agreement, proper institute naming, correct preposition and wording, and improved phrasing for protocol registration and amendment statements). We have also revised the relevant sentences to ensure consistent tense throughout the Methods section and to make the manuscript fully journal-ready.

3. Comment: The conclusion still overreaches

The paper repeatedly acknowledges that pooled AORs are only approximate summaries because studies adjusted for different covariates and that some associations had high heterogeneity. However, the conclusion still moves too far toward intervention language:

“Reducing job stress and prolonged standing…” in the abstract conclusion

“it may be important to improve staffing ratios…” in the final conclusion

For a review based entirely on observational studies, the wording should stay with "were associated with."

“may warrant attention”

“should be interpreted cautiously”

“may inform occupational health planning”

Not with implied preventive effect unless clearly framed as a recommendation rather than evidence of effect.

Response: Thank you for this important comment. We agree that, because the evidence is based entirely on observational studies, the conclusion should not imply preventive or causal effects and should remain focused on associations. In the revised manuscript, we have revised the abstract and final conclusion to replace intervention/causal language with appropriate association-based wording (e.g., using phrasing such as “were associated with" and “may warrant attention").

4. Comment: High-heterogeneity associations are still emphasized too strongly. The manuscript appropriately notes high heterogeneity for job stress and especially prolonged standing. But these factors are still emphasized in the abstract and conclusion almost as if they were robust effect estimates. That is too strong, especially for

Prolonged standing: I² = 91.4%

Job stress: I² = 70.8%

These should remain in the results, but in the abstract and conclusion the authors should make clear that these associations were observed with substantial between-study variability.

Response: Thank you for this valuable comment. We agree that the abstract and conclusion currently emphasize job stress and prolonged standing too strongly relative to the substantial between-study heterogeneity observed. In the revised manuscript, we have revised the abstract and final conclusion to ensure that the pooled findings for prolonged standing and job stress are presented as associations with substantial variability across studies.

5. Comment: The discussion needs more scientific restraint. Some explanatory statements are too assertive or simplistic.

For example, the sex explanation relies heavily on biological reasoning and muscle mass/testosterone differences.

That is possible, but it reads as overly deterministic. The discussion should also acknowledge:

• role differences and task allocation

• domestic workload

• ergonomics

• reporting differences

• workplace context

A more balanced interpretation would be stronger scientifically

Response: Thank you for this valuable comment. In the revised manuscript, we have strengthened the Discussion by providing a more balanced, scientifically restrained interpretation. Specifically, we now acknowledge that the observed sex differences may reflect a combination of factors, including domestic workload

6. Comment: Some claims in the Introduction are overstated or awkwardly phrased

Examples:

• “This variability and scarcity of clear epidemiological understanding deterred the design of diagnostic criteria, the establishment of specialized services, and recruitment in intervention trials.”

This feels exaggerated and probably unsupported.

• “This study is a reliable, evidence-based basis…” should be rewritten more modestly.

The introduction should be tighter and more evidence-led.

Response: Thank you for this constructive comment. We agree that some statements in the introduction were overstated and that the wording should be more modest and evidence-led. In the revised manuscript, we have removed or reframed the exaggerated claims

7. Comment: Methodological concerns

Pooling adjusted odds ratios remains a limitation. The authors now acknowledge that PAORs are approximate because covariates differed across studies. That is good.

Still, the manuscript should be even clearer that

• pooled adjusted effects across heterogeneous models are not equivalent to pooled effects from harmonized adjustment sets

• this reduces comparability and internal validity

• these results are exploratory summaries, not precise quantitative causal estimates

This caution should appear not only in Methods and Table 2's footnote but also briefly in the abstract or conclusion.

Response: Thank you for the comment. We agree that the limitation of pooling adjusted odds ratios requires clear emphasis, not only in the Methods and Table 2 footnote but also in the Conclusion. We therefore revised the Conclusion to explicitly state that the pooled adjusted effects are based on study-specific adjustment sets, which varied across included studies; hence, these pooled estimates are approximate exploratory summaries rather than harmonized-adjustment effects or precise quantitative causal estimates. This wording also clarifies that differences in covariates may reduce comparability and internal validity across studies.

8. Comment: Some factor interpretations are statistically awkward. For work experience:

• PAOR = 1.58 (1.08–2.31), I² = 38.6%, P = 0.196

This appears to mix the heterogeneity p-value with inferential interpretation. The result is statistically significant based on the confidence interval excluding 1, but the narrative should avoid confusing readers by presenting the heterogeneity p-value next to the effect without clarification.

Also, in Table 2 and surrounding text, formatting and presentation should be standardized to avoid ambiguity.

Response: Thank you for the comment. We agree that the way the heterogeneity P-value was presented alongside the pooled adjusted odds ratio (AOR) could be confusing and mix different statistical interpretations. In the revised manuscript, we have removed the heterogeneity P-values from the surrounding text. We have also standardized the formatting and presentation across the relevant sections to improve clarity and avoid ambiguity.

9. Comment: Scope of outcome needs clearer wording

The paper is framed as WMSDs among nurses, but several included studies appear focused on single body regions, especially low back pain or ankle-foot pain.

The authors partly address this by subgrouping according to the number of body areas assessed.

Still, they should state more explicitly that the pooled estimate combines studies with different anatomical scopes, which may affect comparability.

Response: Thank you for this helpful comment. In response, we have revised the manuscript by adding explicit wording in the outcome measurement section to clarify that the included studies differed in anatomical scope (single-region outcomes such as low back pain or ankle/foot pain versus multi-region assessments). We also emphasize that the pooled prevalence estimate therefore combines studies with heterogeneous anatomical definitions, which may affect comparability, and we explain that subgroup analyses were conducted based on the number of body regions assessed to partially address this heterogeneity.

10. Comment: Reference and citation quality should be rechecked. A few citations look weak or mismatched: Reference 30 is a study among bank workers in Ethiopia, yet it is cited for work-related musculoskeletal disorder definition/associated factors in places where a more direct occupational health or nursing source would be stronger.

Some supporting statements in the discussion would benefit from more directly relevant sources and less generic explanation

Response: Thank you for this comment. In the revised manuscript, we rechecked the references and also added additional references.

# Minor issues

11. Comment: Title and abstract wording

The title is acceptable, but the abstract needs line editing for grammar and flow.

For example:

• “Result” should be "Results."

• "Its associated factors” should be “associated factors."

• avoid recommendation-style language in the conclusion

Response: Thank you for the comment. We agree that the abstract requires line editing for grammar, clarity, and flow. In the revised manuscript, we have corrected the identified issues. We have also removed any recommendation-style language in the conclusion, and it should be interpreted as associations with possible between-study variability.

12. Comment: Tables need cleanup

Table 1 and Table 2 are useful, but formatting is rough and should be standardized for journal submission.

Examples:

• inconsistent spacing

• awkward variable labels

• inconsistent capitalization

• some row labels are not reader-friendly

Response: Thank you for this comment. We have reviewed Tables 1 and 2 in the revised manuscript and improved their formatting to meet journal submission standards.

13. Comment: Strengths and limitations

section should be rewritten. The section is useful, but the language is weak and repetitive.

For instance:

• “intensifies transparency” is not natural scientific English

• “negate WMSDs” in the conclusion is incorrect usage

Response: Thank you for this comment. In the revised manuscript, we rewrote the section to improve academic language quality and reduce repetition.

Attachment Submitted filename: Response_to_Reviewers_auresp_3.docx

# Editor report: Decision Letter 3

**DOI:** 10.1371/journal.pone.0354484.r007

**Authors:** Sohel Ahmed

8 Jul 2026

A systematic review and meta-analysis on the epidemiology of work-related musculoskeletal disorders among nurses in Ethiopia

PONE-D-25-47479R3

Dear Dr. Abel Desalegn,

We’re pleased to inform you that your manuscript has been judged scientifically suitable for publication and will be formally accepted for publication once it meets all outstanding technical requirements.

Within one week, you’ll receive an e-mail detailing the required amendments. When these have been addressed, you’ll receive a formal acceptance letter and your manuscript will be scheduled for publication.

An invoice will be generated when your article is formally accepted. Please note, if your institution has a publishing partnership with PLOS and your article meets the relevant criteria, all or part of your publication costs will be covered. Please make sure your user information is up-to-date by logging into Editorial Manager at Editorial Manager® and clicking the ‘Update My Information' link at the top of the page. For questions related to billing, please contact billing support.

If your institution or institutions have a press office, please notify them about your upcoming paper to help maximize its impact. If they’ll be preparing press materials, please inform our press team as soon as possible -- no later than 48 hours after receiving the formal acceptance. Your manuscript will remain under strict press embargo until 2 pm Eastern Time on the date of publication. For more information, please contact onepress@plos.org.

Kind regards,

Sohel Ahmed, BPT, MPT, MDMR

Academic Editor

PLOS One

Additional Editor Comments (optional):

Reviewers' comments:

# Editor report: Acceptance letter

**DOI:** 10.1371/journal.pone.0354484.r008

**Authors:** Sohel Ahmed

PONE-D-25-47479R3

PLOS One

Dear Dr. Demeke,

I'm pleased to inform you that your manuscript has been deemed suitable for publication in PLOS One. Congratulations! Your manuscript is now being handed over to our production team.

At this stage, our production department will prepare your paper for publication. This includes ensuring the following:

* All references, tables, and figures are properly cited

* All relevant supporting information is included in the manuscript submission,

* There are no issues that prevent the paper from being properly typeset

You will receive further instructions from the production team, including instructions on how to review your proof when it is ready. Please keep in mind that we are working through a large volume of accepted articles, so please give us a few days to review your paper and let you know the next and final steps.

Lastly, if your institution or institutions have a press office, please let them know about your upcoming paper now to help maximize its impact. If they'll be preparing press materials, please inform our press team within the next 48 hours. Your manuscript will remain under strict press embargo until 2 pm Eastern Time on the date of publication. For more information, please contact onepress@plos.org.

You will receive an invoice from PLOS for your publication fee after your manuscript has reached the completed accept phase. If you receive an email requesting payment before acceptance or for any other service, this may be a phishing scheme. Learn how to identify phishing emails and protect your accounts at https://explore.plos.org/phishing.

If we can help with anything else, please email us at customercare@plos.org.

Thank you for submitting your work to PLOS ONE and supporting open access.

Kind regards,

PLOS ONE Editorial Office Staff

on behalf of

Dr. Sohel Ahmed

Academic Editor

PLOS One
